# Smart Materials for Dirty Waters: Reversible Aerogels Unlock Closed‐Loop Heavy Metal Remediation

**DOI:** 10.1002/smsc.202500602

**Published:** 2026-03-30

**Authors:** Davide Gentile, Dario Allevi, Massimo Zambito Marsala, Lucrezia Criscuolo, Maurizio Galimberti, Vincenzina Barbera

**Affiliations:** ^1^ Department of Chemistry Materials and Chemical Engineering “G. Natta” Politecnico di Milano Milano Italy; ^2^ ZHAW Zurich University of Applied Sciences IMPE Institute of Materials and Processing Engineering Winterthur Switzerland

**Keywords:** biomass, reversible aerogel, supported catalysts, sustainable chemistry, water remediation

## Abstract

Water remediation technologies urgently require materials that are sustainable, efficient, and regenerable. Here, we report a dynamic covalent aerogel derived from chitosan and formylated lignin, enabling dual functionality in heavy metal adsorption and heterogeneous catalysis. The aerogel is formed through reversible imine bonds between chitosan and aldehyde‐functionalized lignin, synthesized via a mild Reimer–Tiemann reaction. The resulting monoliths exhibit high structural integrity, solvent and pH resistance (pH ≥ 7), and remarkable adsorption capacities for Cu^2+^, Pb^2+^, and Zn^2+^ ions (up to 32.8 mg/g for Cu^2+^). Density functional theory (DFT) calculations reveal tridentate coordination as the dominant binding mechanism. When loaded with Cu^2+^, the aerogels act as effective heterogeneous catalysts for the Chan–Lam crosscoupling reaction in ethanol. They achieve initial yields of up to 92% and retain their catalytic activity over multiple reuse cycles. Importantly, the dynamic covalent framework allows complete recovery and regeneration of all components, underscoring its potential as a circular, bio‐based platform for sustainable water detoxification and green catalysis.

## Graphical abstract

Reversible chitosan–lignin aerogels are developed for efficient heavy metal removal (Cu^2+^, Pb^2+^, and Zn^2+^) from contaminated water, combining high adsorption capacity, recyclability, solvents, and pH stability. The aerogels also demonstrate catalytic activity in Chan–Lam crosscoupling reactions, showcasing their potential for sustainable water detoxification and green catalysis. Density functional theory calculations confirm the synergistic interaction between the aerogels and metal ions, predicting their binding sites.

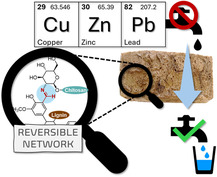

## 1 Introduction

Access to clean water is a fundamental prerequisite for life, on par with air, sunlight, and food. Ensuring its quality and availability remains one of the most critical global challenges of the 21st century, as reflected in Goal 6 of the United Nations 2030 Agenda for Sustainable Development [1]. Yet, as of 2022, more than 2.2 billion people still lack access to safely managed water services [2]. This challenge is magnified by rapid urbanization and industrial expansion, with over half of the global population now living in urban areas— a figure expected to rise to two‐thirds by 2050 [3, 4]. Urban growth increases the demand for freshwater resources [5] and intensifies the release of pollutants into water systems [6].

A major class of contaminants is represented by heavy metals, including Cu^2+^, Pb^2+^, and Zn^2+^, which are discharged from industrial sources such as electroplating, battery manufacturing, and pigment production [7]. These ions are nonbiodegradable and bioaccumulative, posing acute and chronic toxicological risks to both ecosystems and human health [8, 9, 10, 11]. For instance, copper concentrations in industrial effluents can reach up to 50,000 mg/L in plating baths and 3,000 mg/L in wastewater [7], far exceeding the World Health Organization (WHO) limit of 1 mg/L for drinking water [9]. Lead (Pb^2+^), another widespread contaminant, is detected at concentrations up to 50 mg/L in industrial discharge [10], while the WHO guideline for drinking water sets the threshold at 0.01 mg/L [12]. Lead exposure is associated with severe neurological, developmental, and cardiovascular effects [11].

Zinc contamination is similarly pervasive, stemming from galvanization processes, tyre wear particles, and mining residues [13]. Tyres alone are estimated to release over 3 million tons of microplastics annually into the environment [14], many of which contain substantial zinc content. Zn^2+^ ions have been shown to induce oxidative stress and genotoxic effects in aquatic organisms [15]. Regulatory limits are stringent: the US EPA sets a 1.48 mg/L cap for zinc in wastewater [16], while the WHO recommends 0.01 mg/L for surface water [13].

The global burden of untreated wastewater is staggering: an estimated 359.4 billion m^3^ are generated annually, with nearly 80% released untreated into the environment [17]. Unlike organic pollutants, heavy metals are not easily degraded and persist in ecosystems, where they bioaccumulate across trophic levels [18, 19, 20, 21, 22, 23]. Addressing this issue demands the development of efficient, scalable, and sustainable technologies for water remediation.

Conventional strategies such as membrane filtration [24], adsorption on activated carbon [25], and electrocoagulation [26] are effective but often hindered by high costs [27], complex infrastructure, and limited applicability in decentralized or resource‐constrained settings. In addition, the materials used are often not easily regenerable or biodegradable, which limits their application in a circular economy context. Consequently, low‐cost, biocompatible, and regenerative sorbents are increasingly sought after. Biochar and natural minerals such as zeolites have gained attention for their low cost and high metal affinity [28, 29], yet challenges remain in their selectivity, regeneration, and mechanical stability [30].

In addition, conventional aerogel fabrication often requires multistep solvent exchange and energy‐/equipment‐intensive drying processes (e.g. freeze‐drying or supercritical drying), which increases manufacturing costs and can hinder scalability. Recently, hyper‐crosslinked microporous polymers (HCLPs) have attracted attention for heavy‐metal remediation owing to their high surface area and chemical stability. For instance, amine‐functionalized resins can increase the adsorption capacity towards Pb(II) and Cu(II) by more than threefold compared to the nonmodified counterparts [31]. In addition, several representative nonrenewable adsorbents reported in the literature, such as polyethylene glycol diacrylate‐3‐sulfopropyl methacrylate potassium salt (PEGDA‐SMP), show a measurable decrease in adsorption performance after a limited number of regeneration cycles (often within three–five cycles), frequently requiring harsh regeneration protocols (e.g. strong acids) that progressively affect material integrity [32].

Biocomposites composed of biopolymers and organic or inorganic solids [33] offer a promising alternative, combining high adsorption capacity with reusability, tunability, and biocompatibility [19, 20, 34, 35]. Among biopolymers, chitosan (CS) is particularly attractive due to its abundance, biodegradability, and strong affinity for metal ions, conferred by its primary amine and hydroxyl groups [18, 36, 37, 38, 39, 40, 41, 42]. Similarly, lignin (LGN), the second most abundant biopolymer on Earth after cellulose, features a polyphenolic structure rich in active sites (hydroxyl, carbonyl, and methoxy), making it an ideal candidate for pollutant adsorption [43, 44]. Table 1 compares the adsorption capacity and reuse performance of representative chitosan‐ and lignin‐based adsorbents reported in the recent literature, providing a benchmark for the development of renewable and reprocessable remediation technologies.

**TABLE 1:** Comparison of maximum adsorption capacity (*q*_max_) and reusability metrics of representative literature heavy metal adsorbents compared to the current CS/LGN‐CHO aerogel.

Adsorbent material	Target	**Max adsorption capacity,** ***q***_**max**_ **, mg/g**	Reusability	Circularity features	Ref
Amino‐functionalized HCLP	Cu(II), Pb (II)	5.44, 9.88	n.d.	Not renewable	[31]
PEGDA‐SMP hydrogel	Ag(I), Cu(II), Ni(II), Zn(II), Pd(II)	111.01, 92.47, 94.65, 192.11, 87.81	>10 Cycles, Acidic regeneration	Not renewable	[32]
Chitosan/sodium alginate hydrogel	Pb (II), Cu (II), Cd (II)	176.50, 70.83, 81.25	Potentially renewable	Bio‐based material	[45]
Modified cellulose−CS foam	Pb (II), Cd (II), Hg (II), As, Se	75.5, 45.5, 29.0, 66.4, 21.2	n.d.	Bio‐based material	[46]
Chitin‐LGN noncovalent material	Pb (II), Zn (II), Cu (II), Ni (II)	91.74, 82.41, 75.70, 70.41	HNO_3_ can remove adsorbed ions and regenerate the material	Bio‐based material	[47]
Dithiocarbamate‐derived LGN	Cu(II), Zn(II), Ni(II)	98, 78, 67	5 cycles, regeneration *via* HCl	Bio‐based material	[48]
CS‐LGN aerogel	Cu(II), Pb(II), Zn (II)	32.8, 30.2, 27.7	Reversible and regenerable network	Bio‐based material	This work

Chitosan–lignin composites have been previously explored for various applications, including ultraviolet shielding [49, 50], packaging and biomedical uses, and sorption of organic dyes [51]. However, their integration into functional materials often requires high‐energy processing (e.g. shear homogenization) due to their poor miscibility [52]. A promising strategy to overcome these limitations is the formation of covalent dynamic networks through in situ reactions between functionalized lignin and chitosan under mild conditions.

Aerogels, in particular, stand out for their ultralight structure, high porosity, and tunable chemical functionality, making them ideal for adsorption and catalysis [53]. Yet, challenges related to cost, chemical stability, and recyclability still hinder their broader adoption. A significant advancement would be the design of bio‐based aerogels that are efficient at removing metal ions and can be regenerated, disassembled, and incorporated into a circular lifecycle.

**FIGURE 1:**
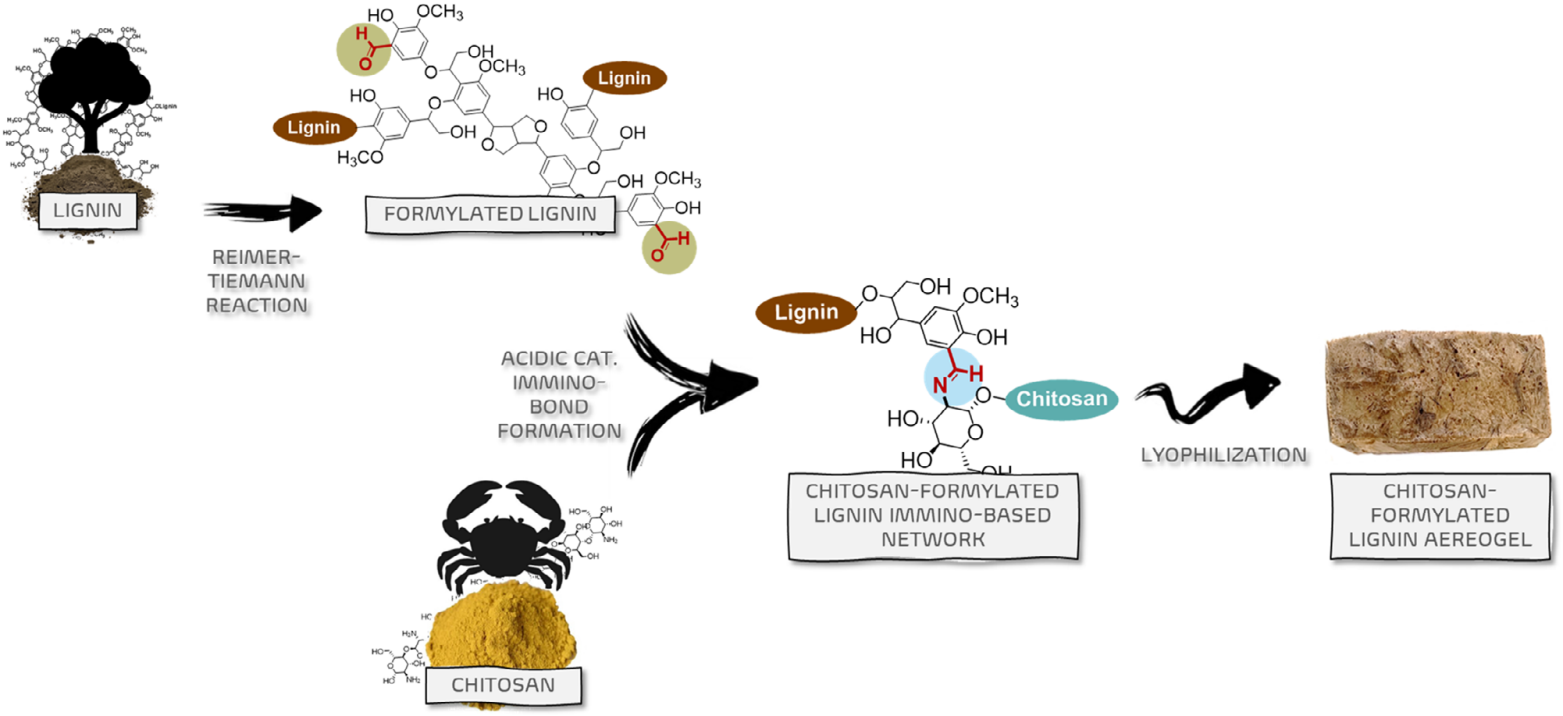
The concept at the base of rational design.

In this work, we report the development of monolithic aerogels based on chitosan and aldehyde‐functionalized lignin (LGN‐CHO), synthesized through a mild and scalable Reimer–Tiemann reaction [54, 55, 56, 57, 58, 59, 60] applied to lignin, demonstrated here for the first time (Figure 1). The aim of functionalizing lignin was to tune the Hansen solubility parameters [61] and reactivity towards chitosan to form stable imine‐linked networks. Although covalent in nature, the crosslinking in this work leverages dynamic covalent chemistry (DCC) through imine bonds, which are reversible under specific stimuli such as pH variations [62]. Compared to permanently crosslinked chitin‐ or lignin‐based adsorbents, where the network cannot be reconfigured once formed [63], this approach is expected to facilitate regeneration under mild conditions and to support repair and reprocessability, consistent with the adaptive nature of DCC systems [47]. Aerogel properties were assessed: solvent resistance, pH stability (≥7), and adsorption capacity for Cu^2+^, Pb^2+^, and Zn^2+^, with removal efficiencies investigated by means of Inductively coupled plasma–optical emission spectroscopy (ICP–OES) analyses. Chelation mechanisms were elaborated through density functional theory (DFT) calculations.

Moreover, the catalytic performance of the Cu^2+^‐loaded aerogels was investigated by performing aqueous Chan–Lam crosscoupling reactions, assessing the yield of the reaction and the catalyst reusability. The objective was the full recovery of the biopolymeric matrix, relying on the reversibility of the covalent network, in line with the principles of closed‐loop material design and molecular sustainability [64, 65].

## 2 Results and Discussion

### 2.1 Preparation and Characterization of LGN‐CHO

**FIGURE 2:**
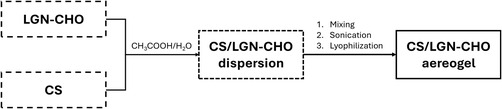
Schematic representation for the preparation of biocomposites between chitosan (CS) and formylated lignin (LGN‐CHO).

As shown in Figure 2  the first step of the research involved the preparation of formylated lignin (LGN‐CHO) through the Reimer–Tiemann reaction. As mentioned in the Introduction section, this reaction enables the introduction of aldehyde groups at the ortho or para positions of phenolic rings using KOH and CHCl_3_ as reagents. In order to determine the appropriate amount of reagents, phosphorus‐31 nuclear magnetic resonance (^31^P‐NMR) was carried out on LGN to quantify the total content of aromatic hydroxyls [66]. Details of the analysis are to be seen in the Supporting Information (Figure S1, Table S1). The reaction was then carried out by respecting the stoichiometry: 1 mol of CHCl_3_ and 3 mol of KOH for each phenolic OH. In brief, LGN and KOH were stirred at room temperature (RT) in a small amount of deionized water to obtain a uniform slurry. CHCl_3_ was added, and the mixture was stirred for 2 h. Deionized water was finally added, pH 7 was achieved by adding acetic acid, and LGN‐CHO was precipitated, collected, washed with deionized water, and dried at room conditions for 48 h.

Tollens’ test was performed as a qualitative analysis on LGN and LGN‐CHO to check the presence of aldehydic groups. Indeed, Tollens’ reagent oxidizes aldehyde groups to the corresponding carboxylic acids and the silver ions are reduced to metals, with the formation of a silver mirror on the test tube (see SI, Figure S2).

The classical silver mirror was observed only when the water suspension was made by using the LGN‐CHO sample. This finding suggests the presence of aldehydic functionalities in the LGN‐CHO sample subjected to the Reimer–Tiemann reaction.

**FIGURE 3:**
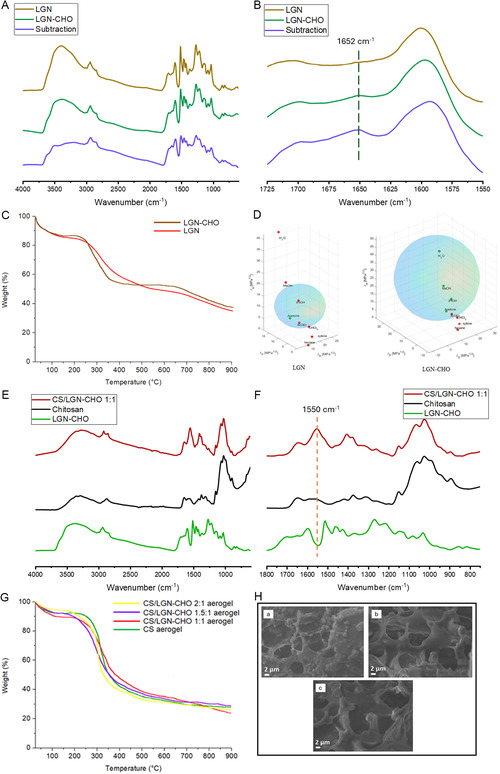
Characterization of biocomposites. (A) FTIR–ATR spectra of LGN (brown line), LGN‐CHO (green line), subtraction spectra (purple line); (B) selection of the 1725–1550 cm^−1^ region: the highlighted peak at 1652 cm^−1^ can be attributed to the C=O stretching of aldehydic groups; (C) TGA thermograms under N_2_ of LGN (red line) and LGN‐CHO (brown line). The thermodegradation profile is similar, indicating that the presence of aldehydic groups did not change the structure; (D) Hansen solubility spheres calculated for LGN and LGN‐CHO. Hansen parameters (Mpa^1/2^): for LGN: *δ*_D_ 18.9, *δ*_P_ 9.4, *δ*_H_ 11.5; for LGN‐CHO: *δ*_D_ 11.1, *δ*_P_ 13.1, *δ*_H_ 28.4. The green circles correspond to the good solvents (within the radius of interaction), and the red circles correspond to the bad solvents (outside the sphere). The solubility profile of formylated lignin shows better suspensibility of the modified lignin. (E) FTIR–ATR spectra of CS/LGN‐CHO 1:1 aerogel (Bordeaux line) compared with the spectra of pristine chitosan (black line) and formylated lignin (green line); (F) selection of the 1800–750 cm^−1^ region: the presence of the imino bond (C=N) can be detected from the presence of the peak at 1550 cm^−1^; (G) TGA thermograms under N_2_ of CS/LGN‐CHO aerogels; (H) SEM micrographs of CS/LGN‐CHO aerogels (a: 1:1 ratio, b: 1.5:1 ratio, and c: 2:1 ratio). Pore sizes have been evaluated through ImageJ by means of 10 measurements, avg pore size: a) 10.880 μm ± 4.166 μm SD, b) 11.253 μm ± 2.102 μm SD, and c) 15.653 μm ± 5.142 μm SD.

Fourier transform infrared spectroscopy–attenuated total reflectance (FTIR–ATR) spectra of LGN and LGN‐CHO are reported in Figure 3A. The broad band around 3390 cm^−1^ is due to the –OH stretching vibration in phenolic and aliphatic units of lignin. The peak at 2915 cm^−1^ is attributed to the stretching of aromatic methoxy groups, while the peak at 2825 cm^−1^ is attributed to the stretching of methyl and methylene groups of the side chains. The signal at 1695 cm^−1^ originated from unconjugated carbonyl/carboxyl stretching, while the one at 1595 cm^−1^ originated from conjugated carbonyl/carboxyl stretching vibrations. The spectral region below 1400 cm^−1^ contains vibrations that are specific [67] to the different monolignol units of lignin. Specifically, the peak at 1270 cm^−1^ is attributed to the C = O vibration in guaiacyl units; the peak at 1203 cm^−1^ is attributed to the combination of the (Ar)–CO–(H, R) and (Ar)–CO–(Ar) vibrations in phenols and aromatic ethers; the peaks at 1145 cm^−1^ are attributed to the C–H stretching of guaiacyl units; the peaks at 1090, 854, and 807 cm^−1^ are attributed to the C–H stretching in syringyl units; and the peak at 1025 cm^−1^ is attributed to the asymmetric stretching of primary aliphatic –OH. The only difference, which can be better highlighted in Figure 3B, between LGN and LGN‐CHO is the peak at 1652 cm^−1^ in LGN‐CHO, which is due to the C=O stretching of aldehydic groups. This is a clear indication that the Reimer–Tiemann reaction led to the formylation of lignin. Furthermore, these findings suggest that the Reimer–Tiemann reaction did not result in any substantial structural modification of lignin.

To quantify the carbonylic functionalities, oximation reaction followed by potentiometric titration were performed on LGN and LGN‐CHO. As mentioned in the Supporting Information, this quantitative analytical technique is carried out with oximating mixture that contains hydroxylamine hydrochloride (NH_2_OH*HCl) and triethanolamine (TEA) in excess. The latter shifts the chemical equilibrium towards a fully oximated product. Details of the analysis have been reported in the Supporting Information (S4). To check the reproducibility of the method, each analysis was performed three times. The results collected after the titration are reported in Table 2.

**TABLE 2:** Carbonyl content determined by oximation reaction.

Sample	%CO	**mmol CO/g**_**sample**_
**LGN**	0.2	0.08
**LGN‐CHO**	1.9	0.62

As mentioned in Table 1, pristine LGN shows a lower amount of carbonyl groups with respect to LGN‐CHO. Specifically, it goes from 0.08 mmol CO/g_sample_ in pristine LGN to 0.62 mmol CO/g_sample_ in LGN‐CHO. This result, in accordance with the Tollens’ test and FTIR–ATR findings, suggests that the formylation of LGN is achieved through the Reimer–Tiemann reaction.

TGA was performed on LGN and LGN‐CHO. Thermograms reported in Figure 3C show that both samples are characterized by a similar profile, with three major degradation steps: *T* < 200°C, 200°C < *T* < 600°C, and *T* > 600°C. The mass losses in the relative temperature ranges are reported in Table 3.

**TABLE 3:** Mass losses of LGN and LGN‐CHO. Data are from TGA analysis.

Sample	Mass loss, %			
	*T* < 200°C	200°C < T < 600°C	*T* > 600°C	Residue
LGN	16	36	16	32
LGN‐CHO	14	31	18	37

Mass losses at *T* < 200°C are attributed to the adsorbed water molecules. The mass loss in the second step, between 200 and 600°C, corresponds to the pyrolysis phenomena typical of lignin segments (syringyl, guaiacyl, and *p*‐coumaryl). The mass loss at *T* > 600°C is due to the decomposition of lignin phenolic units. Both samples present a relevant residue at *T* = 900°C (32% for LGN and 37% for LGN‐CHO) due to the formation of highly condensed aromatic structures. Also, these results suggest that the introduction of aldehydic functionalities in LGN‐CHO did not significantly change the structure of the starting material, LGN.

Hansen solubility parameters *δ*_D_ (dispersion), *δ*_P_ (polar), *δ*_H_ (hydrogen bonding), and the total solubility parameter *δ*_T_ were estimated for LGN and LGN‐CHO. In brief, the materials were suspended in solvents having different solubility parameters, and the stability of the suspension was inspected, as described in the experimental part. The Hansen solubility parameters of the selected solvents are reported in Table S2. The results of the inspection of dispersion stability are qualitatively summarized in Table S3: ‘Good’ or ‘Bad’ means, respectively, that a homogenous dispersion was observed or that the adduct settled. The functionalization of formylated LGN led to the stability of the dispersions of the materials in a good number of solvents. Moving from these observations, the solubility parameters were estimated by adopting the method described in the literature [68, 69, 70, 71]. Solubility spheres, which enclose the good solvents and exclude the bad ones, were generated and are shown in Figure 3D. Values of the solubility parameters and of the radius of the Hansen sphere for LGN and LGN‐CHO are listed in Table 4.

**TABLE 4:** Hansen's solubility parameters for LGN and LGN‐CHO.

Sample	*δ*_D_	*δ*_P_	*δ*_H_	*R*_0_	*δ*_T_ a
**LGN**	18.9	9.4	11.5	8.6	24
**LGN‐CHO**	11.1	13.1	28.4	23.8	33.2

Measure unit: MPa^1/2^.

a *δ*_T_^2^ = (*δ*_H_^2^ + *δ*_P_^2^ +*δ*_T_^2^).

The introduction of –CHO groups in LGN‐CHO modified the solubility parameter of the LGN, which became dispersible in a good variety of solvents. The value of *δ*_T_ was found to be higher for LGN‐CHO. This finding could suggest that the introduction of aldehyde moieties increases the ability of lignin to interact with both aprotic and protic polar solvents.

The Reimer–Tiemann reaction is expected to introduce formyl residues predominantly at the ortho position with respect to phenolic –OH groups. As an electrophilic aromatic substitution, this reaction is directed by the –OH substituent, which exhibits ortho‐ and para‐orienting effects. However, in lignin, the monomeric phenolic units (e.g. para‐coumaryl alcohol and coniferyl alcohol) generally have the para position occupied by aliphatic linkages, leaving only the ortho positions available for formylation.

### 2.2 Preparation and Characterization of CS/LGN‐CHO Aerogels

Biocomposites based on CS and LGN‐CHO were prepared, taking advantage of the ability of the aldehydic groups of LGN‐CHO to react with the primary amines of CS, forming imines. The formation of covalently crosslinked chitosan‐based adducts with the formylated lignin was achieved, thanks to the reaction between the amino groups of chitosan and the aldehydic groups of LGN‐CHO. The composites based on CS/LGN‐CHO adducts were prepared as summarized in the scheme of Figure 2 and described in detail in the experimental part.

The first step was the preparation of CS/LGN‐CHO water dispersions. In brief, CS and LGN‐CHO were first premixed in a mortar with the help of a pestle, at RT, obtaining a physical mixture. Different CS/LGN‐CHO mass ratios were used: 1:1, 1.5:1, and 2:1. Dispersions were then prepared by introducing the CS/LGN‐CHO mixture in a water solution of acetic acid. The obtained suspensions were sonicated for 30 min, at RT, resulting in the formulation of the desired aerogels. A reference sample was also prepared, containing only CS.

FTIR–ATR spectra of CS/LGN‐CHO aerogel are shown in Figure 3, in the 4000–600 cm^−1^ region (Figure 3E) with a focus on the 1800–750 cm^−1^ region (Figure 3F). CS and LGN‐CHO spectra were reported as references.

The spectra present the characteristic signals of both CS and LGN‐CHO. All spectra of the biocomposites present a similar profile. Compared to pristine CS and LGN‐CHO spectra and CS aerogel (reported in detail in the Supporting Information—Figure S3), FTIR–ATR spectra of CS/LGN‐CHO aerogel show the presence of a new band at 1550 cm^−1^. This band is related to the iminic group stretching vibration (C=N) [72, 73] and is not detectable in the spectra of the starting materials nor in the spectra of chitosan aerogel. It is worth emphasizing that the aldehydic signal (1650 cm^−1^) also disappears in the biocomposites spectra. These findings indicate that the aldehydic groups of LGN‐CHO have chemically reacted with the amino groups of CS in the acid media of water suspensions to form a Schiff base and thus a covalent network.

TGA analyses were also conducted on the biocomposites, and the resulting thermograms are shown in Figure 3G. Independently of the mass ratio used for the preparation of aerogels, the curves showed similar profiles, also with respect to CS composites. The thermal behavior did not change significantly. Two major degradation steps are present (T < 200°C and T > 200°C). The first mass loss at T < 200°C is due to the loss of adsorbed water. The second mass loss al T > 200°C is due to the decomposition of the polysaccharidic structure of CS and the aromatic backbone of LGN‐CHO in composites.

Morphological investigations of CS/LGN‐CHO aerogels were performed through scanning electron microscopy (SEM). Micrographs of CS/LGN‐CHO aerogels are in Figure 3H—a, b, and c, for the biocomposites with 1:1, 1.5:1, and 2:1 as the CS/LGN‐CHO mass ratio. LGN‐CHO particles are uniformly distributed in all the aerogels; their degree of agglomeration increases as their concentration increases (from 2:1 to 1:1 as the mass ratio). The CS/LGN‐CHO aerogels reveal a high degree of three‐dimensional crosslinking that makes them spongy and with a large internal surface area. In all cases, a very good interfacial adhesion is evidenced between CS and LGN‐CHO, also increased by the presence of covalent interactions as supported by FTIR–ATR investigations.

### 2.3 Solvent And pH Resistance

Resistance to solvents and to a range of pH was tested for CS/LGN‐CHO aerogels. The resistance to solvents was investigated by using DMF, H_2_O, and *n*‐hexane. Vials containing specimens of aerogels kept for 2 days, at RT, in the different solvents are shown in Figures S4 to S6. The masses of the samples, before and after the storage, are in Tables S4 to S6.

Aerogels revealed an appreciable mass increase in H_2_O, at all the CS/LGN‐CHO ratios. In DMF as the solvent, the nanocomposites swelled and lost their integrity. In *n*‐hexane, stability of the nanocomposites was observed, with a very small solvent absorption.

The pH resistance of CS/LGN‐CHO aerogels was investigated by using HCl, CH_3_COOH, NaHCO_3_, and KOH to modulate the pH. Suspensions of the nanocomposites were visually inspected (see Figures S7 to S9), and nanocomposites taken from the suspensions were weighed. The masses of the CS/LGN‐CHO composites, at 1:1, 1.5:1, and 2:1 ratios, before and after the storage, are in Tables S7 to S9. Aerogels with mass ratios of 1:1 and 1.5:1 were stable in the pH range between 7 and 9. The highest pH value (=14) and the acidic pH (pH = 1 and pH = 4) led to material disruption. The disruption of the aerogels caused by acidic or extremely alkaline pH should not be regarded as an inconvenient issue. Rather, it can be considered a valuable feature that enables recyclability. In fact, imine bonds are reversible covalent bonds that can be formed or cleaved by adjusting the pH of the solution [74, 75]. In fact, imine bonds are reversible covalent bonds that can be formed or cleaved by adjusting the pH of the solution. This characteristic enables the two components of the aerogel to be separated and reused once the product has reached the end of its life. The pH‐responsive disassembly of the aerogels provides experimental evidence of the reversibility of the imine bonds forming the CS/LGN‐CHO network. The selective loss of structural integrity under acidic or extremely alkaline conditions, combined with stability at neutral and basic pH, is fully consistent with the well‐known acid‐catalyzed hydrolysis of imine linkages and supports the dynamic covalent nature of the network.

FTIR analyses, reported in Figure S10 A, of the acid‐treated aerogel, compared with starting materials and aerogel itself clearly shows the disappearance of the C=N signal (1550 cm^−1^) and the presence of the related starting materials (chitosan and, more prominently, formylated lignin). Furthermore, following a neutralization and lyophilization, the CS/LGN‐CHO aerogel treated with a pH 4 solution was rereticulated and analyzed through FTIR and SEM (Figure S10 A,B), and its catalytic activity was evaluated again to confirm system reusability. Those results are reported in Figure S10 C. The fate of the adsorbed metal ions during the regeneration process involves their release into the acidic medium. At pH 4, the protonation of the functional groups in the CS/LGN‐CHO matrix likely triggers the desorption of the metal ions. While not directly quantified in this study, this release is a well‐established phenomenon for pH‐responsive polymeric adsorbents [45, 47, 48] and is supported by the ability of our regenerated material to be successfully refunctionalized for subsequent catalytic cycles. Adsorption‐capacity recovery after regeneration was not quantified in this study; regeneration is discussed as evidence of network re‐formability and functional reuse, while a dedicated quantitative assessment of *q*reg/*q*pristine is beyond the scope of the present work.

### 2.4 Adsorption of Pb^2+^, Cu^2+^, and Zn^2+^ on CS/LGN‐CHO Aerogels

#### 2.4.1 Dynamic Adsorption: Breakthrough Analysis

**FIGURE 4:**
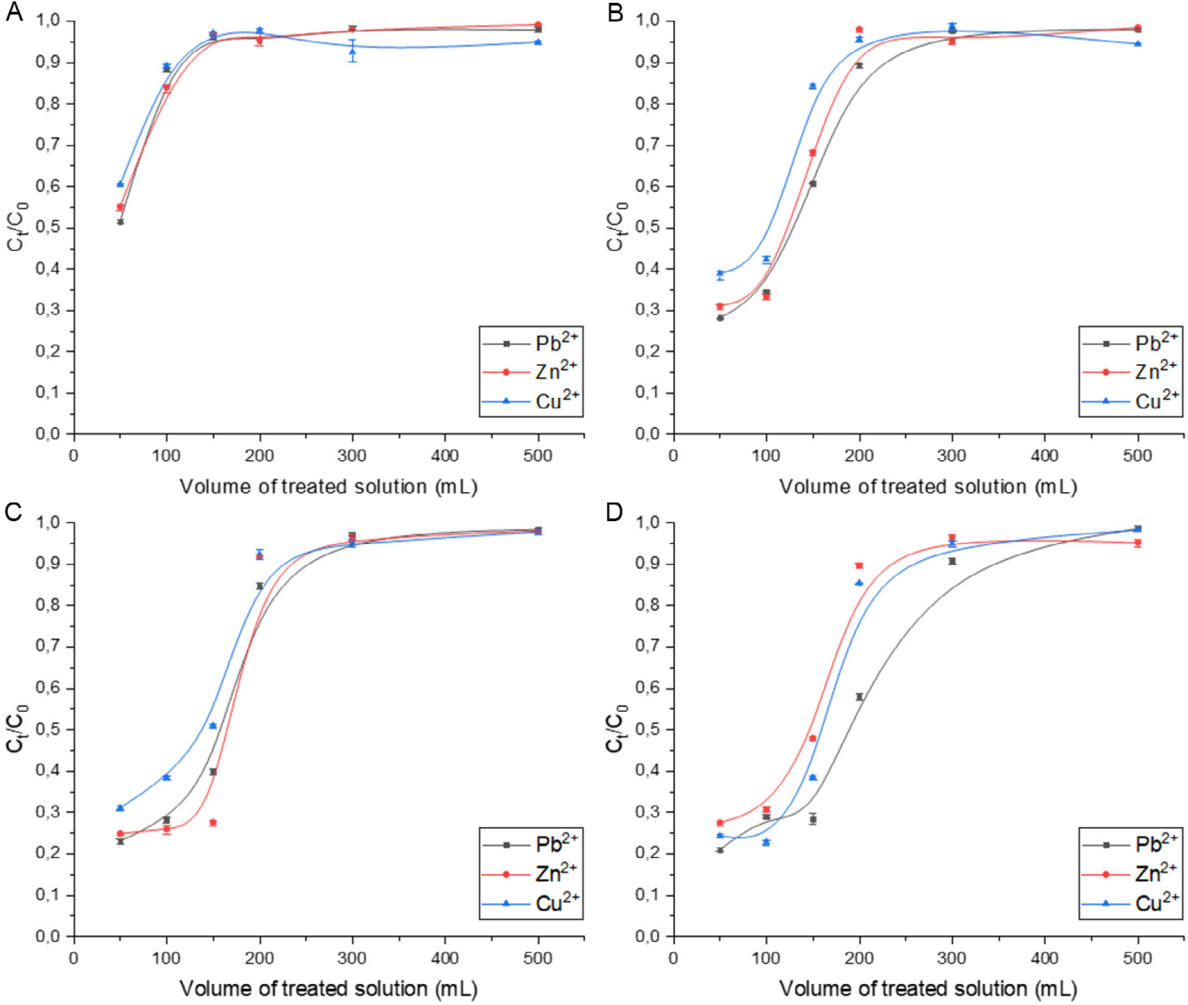
Breakthrough curves (C_t_/C_0_ vs. volume of treated solution) of heavy metal removal using (A) CS; (B) CS/LGN‐CHO aerogel 1:1; (C) CS/LGN‐CHO aerogel 1.5:1; (D) CS/LGN‐CHO aerogel 2:1. C_t_ = concentration of ions in the eluate, C_0_ = concentration of ions in the untreated water (15 mg/L). Error bars in graphical representations indicate the range between the minimum and maximum values recorded for each data point (*n* = 3 independent filtration tests). C_t_/C_o_ values were calculated as the ratio between effluent and influent concentrations.

The ability of CS/LGN‐CHO aerogels to adsorb toxic metals was investigated. Solutions containing Pb^2+^, Cu^2+,^ and Zn^2+^ as metal ions were prepared and filtered by using aerogels. The schematic representation of the filtration system is reported in Figure S11 in Supporting Information. In brief, the solutions with the simultaneous presence of all the ions (15 mg/L each ion) were dripped (with a flow rate of 6 ml min^−1^) into a tube with the aerogel (2 g) as the filter. Samples of 3 mL were collected from the collection beaker, under magnetic stirring, after the following volumes of filtered contaminated solution: 50 mL, 100 mL, 150 mL, 200 mL, 300 mL, and 500 mL. ICP–OES measurements were taken on the collected samples to evaluate the concentration of heavy metals, and breakthrough curves were obtained as the ratio between the concentration of the investigated ions in the eluate and the concentration of the untreated solution (15 mg/L) versus volume of treated solution. Tables S10 to S13 report the registered metal concentration. Figure 4 shows the obtained curves.

The performance of the aerogel monoliths as active filters was investigated by monitoring the concentration of the ions in the eluate over a total treated volume of 500 mL. As reported in Figure 4, the resulting breakthrough curves follow a characteristic saturation trend, where the gradual increase in effluent concentration indicates the progressive exhaustion of the available active sites.

To quantify the adsorption performance under continuous flow, the total dynamic adsorption capacity (q_total_, mg/g) was calculated by integrating the area above the breakthrough curves. This value represents the total mass of metal ions effectively retained by the aerogel monolith until its complete exhaustion. The calculation was performed according to the following equation:

$$q_{\text{total}}=\frac{Q}{m}\int_{0}^{t_{\text{total}}}\left(C_{0}-C_{t}\right)\mathrm{dt}$$

where

• *Q* is the constant flow rate (6 mL/min);
• *m* is the mass of the dry aerogel monolith (2 g);
• *C*_0_ is the initial (influent) metal concentration (15 mg/L);
• *C*_t_ is the effluent concentration at time t (mg/L);
• *t*_total_ is the time required to reach the saturation point.

The integration limits and the operational life of the filters were defined by two critical parameters: the breakthrough points (*V*_b_) and saturation points (*V*_s_). *V*_b_ is defined as the volume of solution treated before the effluent concentration reaches 5% of the initial value (*C*_t_/*C*_0_ = 0.05), marking the limit of immediate filter leach. *V*_s_ is defined as the volume where the effluent concentration reaches 95% of the initial concentration (*C*_t_/*C*_0_ = 0.95), which is representing the complete saturation of the active sites within the 3D framework. Despite the early *V*_b_ occurring due to the high operational flow rate through monolith mass, the steep slope of the curves indicates efficient mass transfer. The integration of the area up to *V*_s_ reveals that the CS/LGN‐CHO 2:1 composite significantly extends the filter's life compared to pristine CS, tripling the operational capacity for Pb^2+^. This dynamic behavior is consistent with the maximum adsorption capacities measured in batch tests (Table S14) and the high interaction energies calculated via DFT, revealing a great affinity for copper (Δ*E*_int_ = −377.34 kcal/mol), which confirm Cu^2+^ as the most strongly coordinated cation within the tridentate ONO pocket.

Resultant *V*_b_ and *V*_s_ points, calculated for each ion and each aerogel, are reported in Table 5.

**TABLE 5:** Breakthrough and saturation points for CS and CS/LGN‐CHO aerogels.

Ions	CS		CS/LGN‐CHO aerogel 1:1		CS/LGN‐CHO aerogel 1:1,5		CS/LGN‐CHO aerogel 1:2	
	(*V*_b_)	(*V*_s_)	(*V*_b_)	(*V*_s_)	(*V*_b_)	(*V*_s_)	(*V*_b_)	(*V*_s_)
Pb^2+^	<50 mL	138.8 mL	<50 mL	263.8 mL	<50 mL	283.9 mL	<50 mL	410.9 mL
Zn^2+^	<50 mL	141.6 mL	<50 mL	195.4 mL	<50 mL	279.0 mL	<50 mL	281.2 mL
Cu^2+^	<50 mL	>500 mL	<50 mL	>500 mL	<50 mL	311.8 mL	<50 mL	295.5 mL

Generally, all the composites showed good affinity for the simultaneous removal of the bivalent cations present in solution. The action of active sites on both CS (such as hydroxyl and free primary amine groups) and LGN‐CHO (such as hydroxyl, carbonyl, and methoxy groups) was effective in retaining metal pollutants.

#### 2.4.2 Static Adsorption: Maximum Capacity

Batch adsorption tests were performed to define the thermodynamic limit of the materials. Aerogels were posed at an high initial concentration (100 ppm) for 12 h to ensure complete equilibration. The results, summarized in Table S14, show maximum adsorption capacities (q_max_):

Cu^2+^ capacity: Reached a peak of 32.8 mg/g for the 2:1 aerogel.

Pb^2+^ and Zn^2+^ capacity: Achieved up to 30.2 mg/g and 27.7 mg/g, respectively.

### 2.5 Density Functional Theory Calculations

**FIGURE 5:**
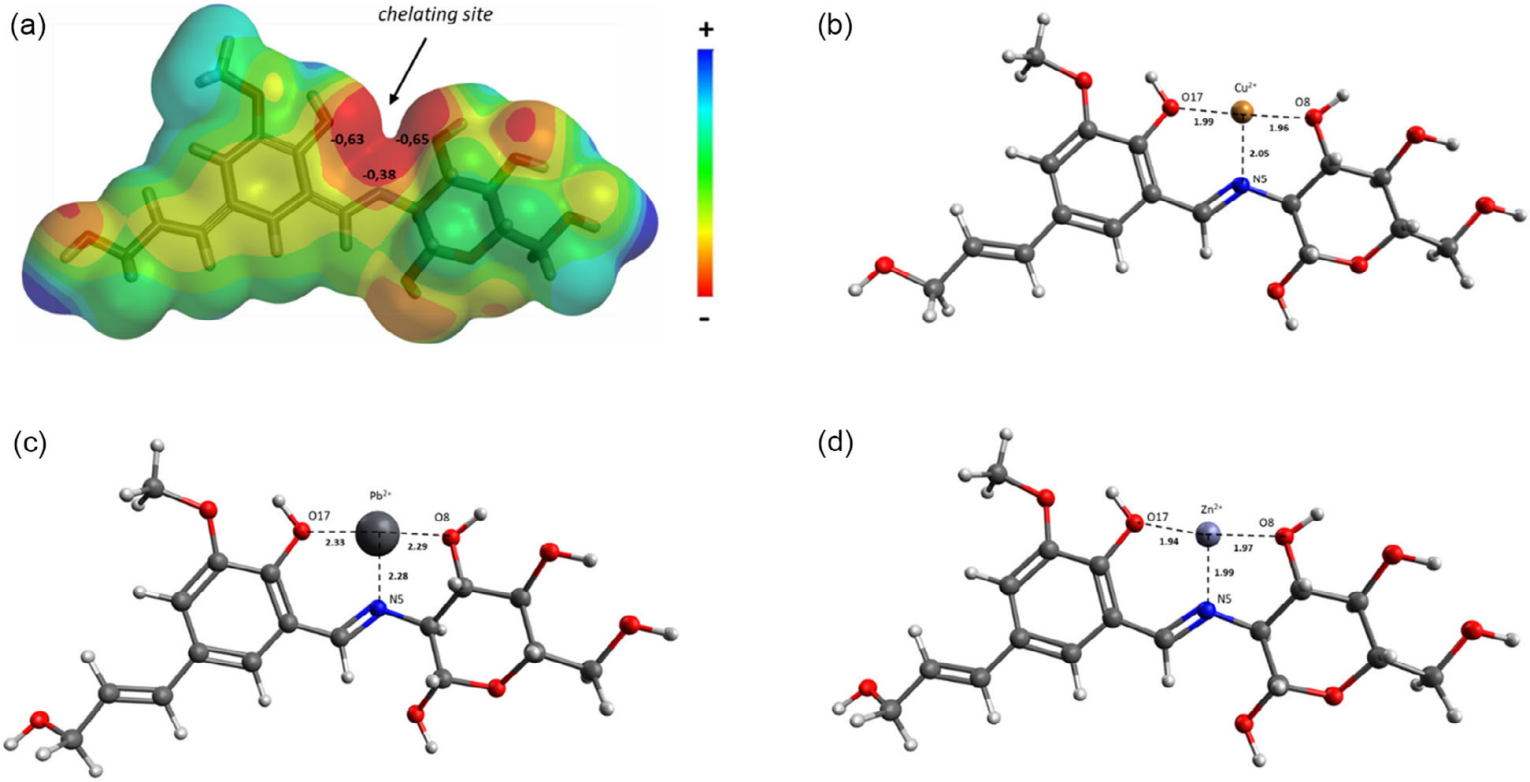
(a) Molecular electrostatic potential surfaces of model compounds: CS/LGN‐CHO. (b) optimized structures of complex CS/LGN‐CHO‐Cu^2+^, (c) complex CS/LGN‐CHO‐Pb^2+^, and (d) complex CS/LGN‐CHO‐Zn^2+^.

To investigate the interaction of metals with aerogels, DFT calculations were performed. The most stable complexes were fully optimized at the DFT level of theory using a B3LYP and LANL2DZ basis set, and a full analysis of the natural bond orbitals (NBOs) was performed. The DFT calculation was performed using the Gaussian09 program suite [76]. The optimized geometry of the CS/LGN‐CHO complex with ions is shown in Figure 5. Since CS and LGN‐CHO are high polymers and the unit structures are very large, the initial Schiff base structure for DFT calculation has been simplified to improve calculation efficiency. In this case, the Schiff basis proposed for DFT calculations derives from the condensation between the aldehyde of the derived coniferyl alcohol and D‐glucosamine, that is, from the main monomers of lignin and chitosan.

The molecular electrostatic potential surfaces (MEPS) (Figure 5a) is a very useful property, as it provides information on the local reactivity of the molecules, localizing the density of electrons present. Through the analysis of MEPS, it is possible to predict possible sites of complex formation [77]. The points of greatest reactivity of the molecule can be observed via MEPS in the regions with the most intense colors, where blue indicates a partially positive region and the more orange or red colors are partially negative regions.

The MEPS map demonstrated that the ONO triad is the main active adsorption site for Pb^2+^, Zn^2+^, and Cu^2+^ ions, forming a basic tridentate ligand system containing a metal‐accessible coordination site. Based on the net Mulliken charge analysis (Figure 5), the negative charges of the O6‐N1‐O2 triad were calculated to be −0.63 for O6, −0.38 N1, and −0.65 for O2, respectively. These results indicate the three atoms could act as electron donors to interact with Pb^2+^, Cu^2+^, and Zn^2+^ ions.

In Figure 5, it was observed that the presence of the metal ion generates distortions in the structure, mainly due to the electrostatic attraction or repulsion forces involved.

Although the metals were initially all positioned in the same position after the interaction calculations, some of them gave rise to different behaviors due to the sizes of the cations and the energies involved (Table 6). However, only after analysing adsorption mechanisms and the energies of the chelating processes, it is possible to clarify the relationship between the species. The interaction energies were calculated and are shown in Table 6.

**TABLE 6:** Electronic interaction energies (E_int_) and interaction length for the studied complexes.

Complex	Type interaction	Interaction length, Å	**ΔE_int_, kcal mol** ^**−1**^
CS/LGN‐CHO‐Cu^2+^	O8‐Cu^2+^	1.96	−377.34
	N5‐Cu^2+^	2.05	
	O17‐Cu^2+^	1.99	
CS/LGN‐CHO‐Pb^2+^	O8‐Pb^2+^	2.29	−336.34
	N5‐Pb^2+^	2.28	
	O17‐Pb^2+^	2.33	
CS/LGN‐CHO‐Zn^2+^	O8‐Zn^2+^	1.94	−314.29
	N5‐Zn^2+^	1.99	
	O17‐Zn^2+^	1.97	

The metal–nitrogen distances in the Cu^2+^, Pb^2+^, and Zn^2+^ complexes are 2.05, 2.28, and 1.99 Å, respectively, while the bond distances between the metal ions and oxygen atoms in the complexes are similar between the Zn^2+^ and Cu^2+^ atoms, and the distances with Pb^2+^ are higher. Furthermore, the interaction energy (E_int_ = E complex − E 1 − E Me^2+^) was in favor of Cu^2+^, with an E_int_ of −377.34 kcal/mol, compared to Pb^2+^ and Zn^2+^ (E_int_ −336.34 and −314.29 kcal/mol, respectively).

Although Pb^2+^ causes a greater distortion of the chelating site, due to a greater volume of the ion, E_int_ is greater than Zn^2+^, in agreement with experimental data.

To gain insight into the electronic nature of metal–ligand bonding in the CS/LGN‐CHO complexes of Cu^2+^, Pb^2+^, and Zn^2+^, we performed Wiberg bond index (WBI) and NBO analyses. The WBI results (Table 7) revealed notable differences in covalent character across the metal centers. Zn^2+^ exhibited the highest WBI values with all donor atoms (O8: 0.229, N5: 0.245, and O17: 0.217), indicating stronger covalent interactions likely favored by its closed‐shell d^10^ configuration and effective orbital overlap. Pb^2+^ displayed intermediate values, particularly elevated with nitrogen (N5: 0.289), reflecting polarizable bonding supported by the involvement of diffuse 6p orbitals. Cu^2+^ showed the lowest indices (~0.04–0.05), consistent with weaker covalency and a greater dependence on electrostatic interactions. Complementary NBO second‐order perturbation analysis (Tables S15–S17) revealed donor–acceptor interactions from lone pairs on N and O atoms into vacant metal‐centered orbitals. Zn^2+^ complexes showed the most stabilizing interactions, with E(2) values reaching 61 kcal/mol, followed by Pb^2+^ with energies up to 51 kcal/mol. Cu^2+^, while exhibiting lower E(2) values (<16 kcal/mol) still forms energetically favorable interactions. Notably, the trends in WBI does not directly correlate with total interaction energies (E_int_), which were found to be highest for Cu^2+^. This suggests that while Zn^2+^ bonds are more covalent, Cu^2+^ achieves greater overall stabilization through geometry and electrostatic contributions. Frontier molecular orbitals, specifically the highest occupied molecular orbital (HOMO) and the lowest unoccupied molecular orbital (LUMO), offer valuable insights into the electronic structure and reactivity of coordination systems. The HOMO and LUMO energies of the model compounds (CS/LGN‐CHO, CS/LGN‐CHO–Cu^2+^, CS/LGN‐CHO–Pb^2+^, and CS/LGN‐CHO–Zn^2+^) were obtained from three‐dimensional orbital plots, and the corresponding HOMO–LUMO energy gaps (ΔE) were calculated at the B3LYP/LANL2DZ level of theory (Figure S12). The calculated Δ*E* values for CS/LGN‐CHO, CS/LGN‐CHO–Cu^2+^, CS/LGN‐CHO–Pb^2+^, and CS/LGN‐CHO–Zn^2+^ are 4.26 eV, 3.83 eV, 1.80 eV, and 2.04 eV, respectively. Introducing metal ions significantly reduces the energy gap compared to the pristine CS/LGN‐CHO model, indicating increased electronic delocalization upon coordination.

**TABLE 7:** Wiberg bond indices calculated between the indicated complexes atoms Cu^2+^, Pb^2+^, and Zn^2+^.

Atom	O8	N5	O17
**Cu** ^**2+**^	0.052	0.043	0.0405
**Pb** ^**2+**^	0.153	0.289	0.187
**Zn** ^**2+**^	0.229	0.245	0.217

It has been established that, despite the fact that Cu^2+^, Pb^2+^, and Zn^2+^ interact with the CS/LGN‐CHO framework through the same ONO tridentate coordination motif, their binding energies differ as a result of fundamental inorganic chemical factors, namely, ionic radius, charge density, and polarizability. Pb^2+^, distinguished by its notably larger ionic radius and reduced charge density, generates extended metal–donor distances and elicits a more pronounced distortion of the ONO coordination pocket, as evidenced by the optimized geometries presented in Table 6. Zn^2+^, with its smaller ionic radius and higher charge density, exhibits shorter coordination distances, thereby favoring stronger orbital overlap with the donor atoms. In the context of ionic size, Cu^2+^ can be considered an intermediate case. However, it benefits from a favorable balance between charge density and geometric accommodation within the ONO site, which results in the most favorable overall interaction energy (ΔE_int_). Despite this, its degree of covalent character is lower compared to Zn^2+^. Pb^2+^, while exhibiting greater polarizability, partially compensates for its diminished charge density through enhanced electrostatic stabilization, resulting in intermediate binding strength (Table S19).

These geometric considerations are complemented by electronic structure descriptors. NBO charge analysis (Supporting Information) confirms electron density transfer from the ONO donor atoms to the metal center upon coordination, while Wiberg bond indices (Table 7) indicate a larger covalent contribution for Zn^2+^ compared to Cu^2+^ and Pb^2+^. It is noteworthy that the trends in bond order do not directly correlate with interaction energies, thus emphasizing the predominant role of electrostatic contributions—modulated by ionic radius and charge density—in determining the binding strength of Cu^2+^, whereas covalent contributions become more significant for Zn^2+^.

### 2.6 Chan–Lam Cross‐Coupling Reactions by CS/LGN‐CHO‐Cu^2+^ Catalyst

The Chan–Lam reaction is an efficient strategy for the formation of C–N bonds via oxidative coupling between boronic acids and heteroatom‐containing nucleophiles. Heterogeneous copper‐based catalysts, incorporated into polymeric matrices, inorganic supports, or nanostructured materials offer a sustainable alternative to homogeneous systems. These catalysts combine high catalytic efficiency with easy recovery and reusability. Moreover, they enable the reaction to proceed under mild conditions, often in green solvents and with good functional group tolerance. These features make them promising tools for sustainable organic synthesis and industrial applications.

The CS/LGN‐CHO‐Cu^2+^ catalyst was then evaluated for its ability to promote the Chan–Lam crosscoupling reaction, which enables the formation of C–N bonds through the oxidative coupling of arylboronic acids with nitrogen‐based nucleophiles. This heterogeneous catalytic system is based on a biocomposite material in which Cu^2+^ ions are immobilized within a covalently crosslinked aerogel matrix via imine bonds composed of chitosan and formylated lignin (LGN‐CHO).

**FIGURE 6:**
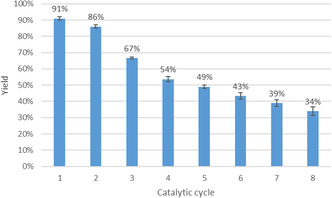
Reusability of the CS/LGN‐CHO–Cu^2+^ aerogel catalyst in the Chan–Lam crosscoupling reaction. Yield refers to the isolated yield of the crosscoupling product obtained after each catalytic cycle. Error bars represent the standard deviation (SD) calculated from three independent experimental replicates.

**SCHEME 1:**
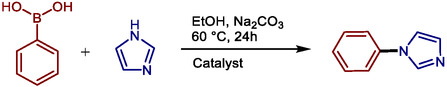
Chan–Lam crosscoupling reaction catalyzed by CS/LGN‐CHO‐Cu^2+^.

To investigate the catalytic activity of the complex for the Chan–Lam reaction, experiments were carried out using imidazole and phenylboronic acid as model substrates, as shown in Scheme 1. The results are shown in Figure 6. The reaction was performed by stirring a mixture of phenyl boronic acid (1.0 mmol), imidazole (1.0 mmol), and Na_2_CO_3_ (1.0 mmol) in ethanol at 60°C for 24h. The reaction proceeded efficiently in the presence of 20 wt% of the CS/LGN‐CHO‐Cu^2+^ catalyst, affording the desired *N*‐arylated product in good yield, as confirmed by NMR analysis. After completion, the reaction mixture was cooled to RT for 1 h, after which the catalyst was recovered by filtration and thoroughly washed with ethanol to remove residual reactants or products. After that, the washed catalyst was put again in a reaction tube with new starting materials. The catalyst demonstrated appreciable activity upon the first reuse; however, a gradual decline in reaction yield was observed over successive cycles (Figure 6).

**FIGURE 7:**
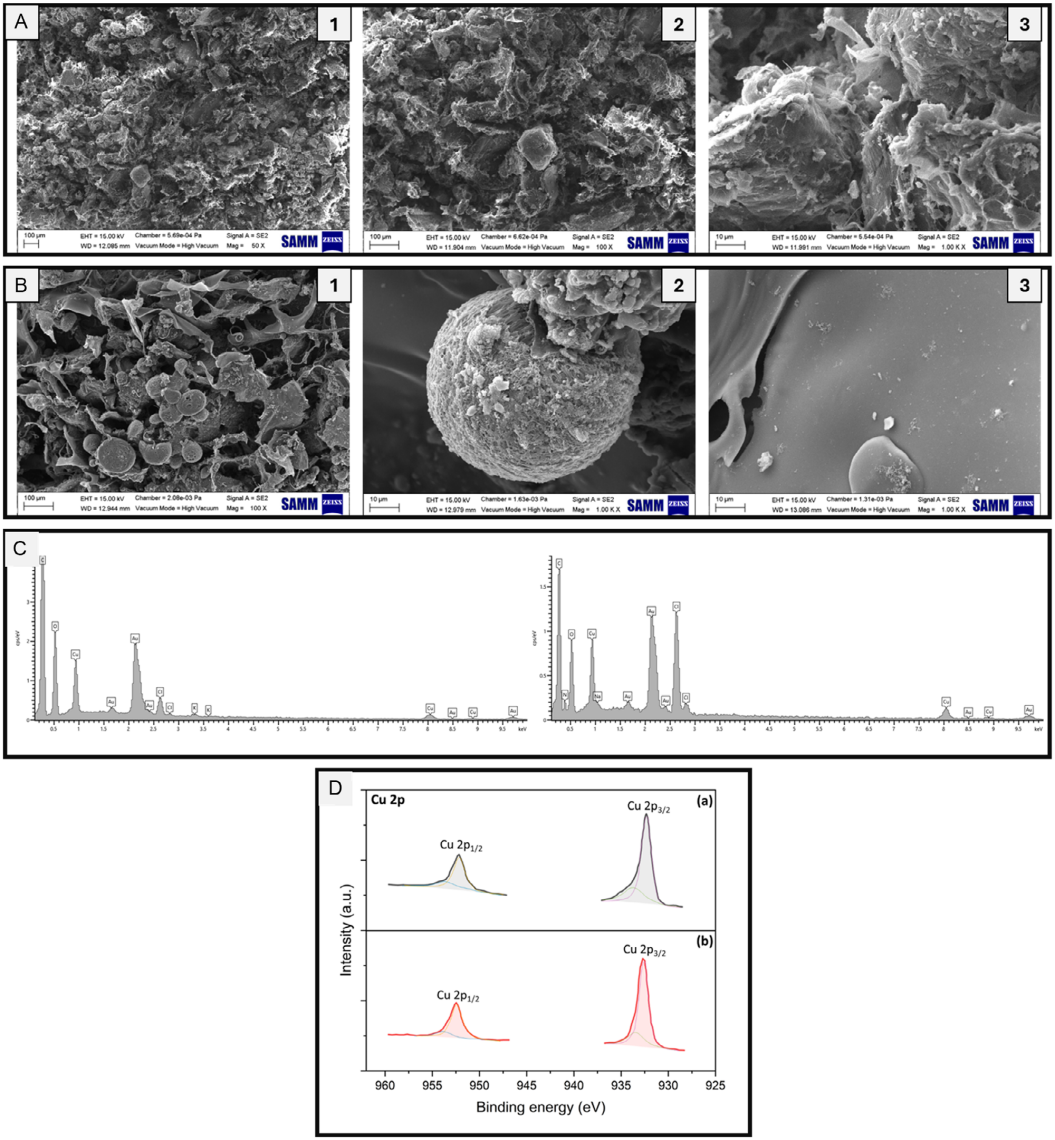
SEM micrographs of: (A) CS/LGN‐CHO aerogels after Cu^2+^ loading (**A1‐3** represents the aerogel under different magnification); and (B) CS/LGN‐CHO aerogels after the eighth cycle of reaction (**B1‐3** represents different areas and structures of the aerogel). (C) Representative EDS spectra of the sample surface (left: after Cu^2+^ loading; right: after the eighth cycle of reaction), showing the presence of Cu, Cl, and O. The Au peaks are attributed to the conductive coating used for SEM imaging. (D) High‐resolution Cu 2p XPS spectra of the catalyst recorded (a) before and (b) after the catalytic cycles.

SEM analysis shows that the loss of catalytic activity could be correlated with a loss in the structural integrity of the aerogels, as can be seen in Figure 7A‐B: after the eighth cycle, the structure appears composed of sheets and spherical aggregates, with a significant loss of the original porous network. The energy‐dispersive X‐ray spectroscopy (EDS), coupled with SEM analysis (Figure 7C), instead shows that the Cu leaching is negligible. To further investigate the catalytic activity of this aerogel, XPS analysis was also performed on the same samples. The as‐prepared catalyst displayed a Cu surface content of 0.4%, confirming the successful incorporation of the metal. As shown in Figure 7D‐a, the high‐resolution Cu 2p spectrum exhibits two characteristic peaks at binding energies of 932.5 eV and 952.4 eV, corresponding to Cu 2p3/2 and Cu 2p1/2, respectively [78]. The relatively low binding energies suggest strong electronic interaction between Cu species and the carbon framework, indicative of electron transfer from the carbon support to Cu. Comparison of the Cu 2p spectra recorded before and after the catalytic cycles reveals a slight decrease in Cu surface content to 0.3%, suggesting partial metal loss during operation. However, as shown in Figure 7D‐b, no significant changes in peak shape or overall spectral features are observed, indicating good chemical and structural stability of the Cu species. This stability can be attributed to the protective aerogel framework, which helps preserve the Cu active sites during operation, as well as to the strong electronic coupling between Cu and the organic support. A minor positive shift of both Cu 2p peaks is observed after catalysis, with the Cu 2p3/2 and Cu 2p1/2 peaks centered at 932.9 eV and 952.7 eV, respectively, suggesting a slight reduction in charge transfer and a subtle changes in the Cu coordination environment following catalytic use.

The aerogel's porous structure further facilitates reagent diffusion and access to active sites. These features, combined with the use of renewable biopolymers or harsh conditions, position the CS/LGN‐CHO‐Cu^2+^ catalyst as an attractive alternative to traditional homogeneous systems for green synthetic applications. Importantly, the catalyst demonstrated notable recyclability, retaining its activity across multiple reaction cycles, highlighting its operational stability and economic viability.

The recycling experiments reported in Figure 6 are intended as a proof‐of‐concept demonstration of the catalytic feasibility of the CS/LGN‐CHO‐Cu^2+^ aerogel. The catalyst shows appreciable activity over multiple runs; however, a progressive decline in yield is observed upon reuse, reaching 35% by the eighth cycle. This decrease is attributed to gradual structural degradation of the aerogel framework, as supported by post‐reaction morphological characterization, which compromises the integrity of the porous network under repeated catalytic conditions.

Post‐regeneration through the pH‐responsive protocol previously outlined, the CS/LGN‐CHO aerogels were subsequently subjected to testing in the Chan–Lam crosscoupling reaction, utilizing the same conditions as employed for the pristine materials. The catalytic activity of the regenerated aerogels, as illustrated in Figure S10 C (Supporting Information), demonstrates the retention of functional catalytic performance after regeneration, thereby substantiating the viability of a closed‐loop regeneration–reuse strategy.

## 3 Conclusions

Aerogels based on CS and LGN‐CHO are effective materials for removing from water ions of toxic metals such as Cu^2+^, Zn^2+^, and Pb^2+^. The Reimer–Tiemann reaction was for the first time successfully performed on lignin, obtaining formylated LGN. CS/LGN‐CHO bionanocomposites were then prepared by mixing CS and LGN‐CHO with mortar and pestle and then lyophilizing stable water dispersion of CS/LGN‐CHO. Monolithic aerogels were obtained, showing good resistance to solvents (water and n‐hexane, while DMF led to material disruption) and pH variations (pH range between 7 and 9). FTIR analysis revealed the formation of a covalent iminic bond between CS and LGN‐CHO at the basis of a continuous CS/LGN network. Aerogel regeneration was performed after acid treatment under mild conditions demonstrating that the system is a closed‐loop system. These results support pH‐triggered cleavage of the imine crosslinks and macroscopic re‐formability of the network, while chain‐level integrity of the recovered components was not evaluated in this study. The aerogels were able to adsorb the divalent ions of Cu, Pb, and Zn up to a level of 32.8 mg/g, 30.2 mg/g, and 27.7 mg/g, respectively. The DFT analysis revealed the synergy between CS and LGN‐CHO for favoring the efficient adsorption of the metal ions. Indeed, a tridentate chelation frames the cations, and the chelating nitrogen atoms belong to CS and to the iminic group formed by the reaction of CS and LGN‐CHO. The CS/LGN‐CHO–Cu^2+^ aerogels were shown to function as effective heterogeneous catalysts for the Chan–Lam cross‐coupling reaction, displaying good catalytic activity in aqueous media at moderate temperatures. The catalytic system exhibited high initial catalytic activity, achieving a yield of up to 92%, and remained catalytically active over several consecutive reuse cycles. This demonstrates its applicability as proof of concept.

This preliminary study in heterogeneous catalysis opens a number of promising avenues for extending the approach to other cross‐coupling reactions. Finally, the regenerated CS/LGN‐CHO aerogels were shown to retain catalytic activity in the Chan–Lam cross‐coupling reaction after regeneration, further demonstrating the feasibility of a closed‐loop regeneration and reuse strategy.

## Author Contributions

**Davide Gentile**: methodology, formal analysis, investigation, data curation, writing – review and editing. **Dario Allevi**: validation, writing – original draft, writing – review and editing. **Massimo Zambito Marsala**: methodology, formal analysis, investigation, data curation, writing – original draft. **Lucrezia Criscuolo**: formal analysis, writing – original draft; **Maurizio Galimberti**: conceptualization, writing – original draft, writing – review and editing, funding acquisition. **Vincenzina Barbera**: conceptualization, methodology, validation, investigation, writing – original draft, writing – review & editing, supervision, project administration, funding acquisition. All authors have read and agreed to the published version of the manuscript.

## Supporting Information

Additional supporting information can be found online in the Supporting Information section. The authors have cited additional references within the Supporting Information [61, 66, 68, 71, 76, 79, 80, 81, 82, 83]. **Supporting Fig. S1**: ^31^P‐NMR spectrum of LGN. **Supporting Fig. S2**: Tollens’ test results of LGN‐CHO and LGN water suspensions. **Supporting Fig. S3**: FTIR–ATR spectra of CS (black line), CS aerogel 1 (orange line), and CS aerogel 2 (green line). **Supporting Fig. S4**: Solvent resistance of CS/LGN‐CHO 1:1 aerogel. **Supporting Fig. S5**: Solvent resistance of CS/LGN‐CHO 1.5:1 aerogel. **Supporting Fig. S6**: Solvent resistance of CS/LGN‐CHO 2:1 aerogel. **Supporting Fig. S7**: CS/LGN‐CHO 1:1 aerogel in water at different pH. **Supporting Fig. S8**: CS/LGN‐CHO 1.5:1 aerogel in water at different pH. **Supporting Fig. S9**: CS/LGN‐CHO 2:1 aerogel in water at different pH. **Supporting Fig. S10**: A. FTIR spectra of the acid‐treated aerogel CS/LGN‐CHO 1:1; A1. Regenerated aerogel; A2. Treated aerogel (pH 4, 30 days); A3. Untreated aerogel; A4. Chitosan; A5. Formylated lignin; B. SEM micrograph of: untreated aerogel (B1); regenerated aerogel (B2); C. Catalytic performances of the regenerated aerogel. **Supporting Fig. S11**: Filtration system adopted for heavy metals removal from contaminated water solution. **Supporting Fig. S12**: Representation of the HOMO, LUMO, and Egap of: (A) CS/LGN‐CHO; (B) optimized structures of the complex CS/LGN‐CHO‐Cu^2+^, (C) the complex CS/LGN‐CHO‐Zn^2+^, (D) the complex CS/LGN‐CHO‐Pb^2+^. **Supporting Table S1**: Hydroxyl content of lignin determined through ^31^P‐NMR. **Supporting Table S2**: Hansen solubility parameters for selected solvents and results of inspection of dispersions of LGN and LGN‐CHO after 1 week. **Supporting Table S3**: Results of inspection of dispersions of LGN and LGN‐CHO in various solvents. **Supporting Table S4**: Weights of CS/LGN‐CHO 1:1 aerogels. **Supporting Table S5**: Weights of CS/LGN‐CHO 1.5:1 aerogel. **Supporting Table S6**: Weights of CS/LGN‐CHO 2:1 aerogel. **Supporting Table S7**: Weights of CS/LGN‐CHO 1:1 aerogel. **Supporting Table S8**: Weights of CS/LGN‐CHO 1.5:1 aerogel. **Supporting Table S9**: Weights of CS/LGN‐CHO 2:1 aerogel. **Supporting Table S10**: Concentration of heavy metals detected using CS aerogel as adsorbent. **Supporting Table S11**: Concentration of heavy metals detected using CS/LGN‐CHO 1:1 aerogel as adsorbent. **Supporting Table S12**: Concentration of heavy metals detected using CS/LGN‐CHO 1.5:1 aerogel as adsorbent. **Supporting Table S13**: Concentration of heavy metals detected using CS/LGN‐CHO 2:1 aerogel as adsorbent. **Supporting Table S14**: Results of the batch absorption test. **Supporting Table S15**: Second‐order perturbation theory analysis of Fock matrix in NBO basis. NBO Interactions (E(2) > 1.0 kcal/mol) for Cu^2+^ complex. **Supporting Table S16**: Second‐order perturbation theory analysis of Fock matrix in NBO basis. NBO Interactions (E(2) > 1.0 kcal/mol) for Pb^2+^ complex. **Supporting Table S17**: Second‐order perturbation theory analysis of Fock matrix in NBO basis. NBO Interactions (E(2) > 1.0 kcal/mol) for Zn^2+^ complex. **Supporting Table S18**: Cartesian coordinates of all investigated structures (Angstroms). **Supporting Table S19**: Atomic charges (Hirshfeld population analysis) for ONO donor atoms and metal centers in CS/LGN‐CHO complexes.

## Funding

Made in Italy—Circular and Sustainable (MICS) Extended Partnership funded by the European Union Next‐Generation EU (Piano Nazionale di Ripresa e Resilienza (PNRR)—Missione 4, Componente 2, Investimento 1.3—D.D. 1551.11‐10‐2022, PE00000004); the Circular Economy Lab for Life Sciences‐CELLS within the MUSA—Multilayered Urban Sustainability Action—project, funded by the European Union—NextGenerationEU, under the National Recovery and Resilience Plan (NRRP) Mission 4 Component 2 Investment Line 1.5: Strengthening of research structures and creation of R&D 'innovation ecosystems', set up of 'territorial leaders in R&D'; MadABio project—funded by European Union—Next Generation EU within the PRIN 2022 PNRR program (D.D.1409‐14/09/2022 Ministero dell’Università e della Ricerca).

## Conflicts of Interest

The authors declare no conflicts of interest.

## Supporting information

Supplementary Material

## Data Availability

All data supporting the findings of this study, including raw and processed experimental datasets (NMR spectra; Tollens’ tests; carbonyl group content determination; Hansen solubility parameters; dispersion in different solvents; FTIR‐ATR spectra; solvent and pH resistance; and DFT calculations), are available within the published article and its supplementary information. Additional raw data has been deposited at https://zenodo.org/records/18920425. Further information can be requested from the corresponding author.
