# Supplementary material for: Smart Materials for Dirty Waters: Reversible Aerogels Unlock Closed‐Loop Heavy Metal Remediation
Source: Small Sci. 2026 Mar 30;6(4):e202500602. doi: 10.1002/smsc.202500602 (PMC13154914; doi:10.1002/smsc.202500602)
Supplement: Supplementary file 1 — Supplementary Material [file SMSC-6-e202500602-s001.pdf]

SUPPORTING INFORMATION

Smart Materials for Dirty Waters: Reversible Aerogels Unlock Closed-Loop Heavy Metal Remediation

Davide Gentile,[1†] Dario Allevi, [1] Massimo Zambito Marsala,[† 1,2] Lucrezia Criscuolo, [1] Maurizio Galimberti[1] and Vincenzina Barbera[1*]

[1]Department of Chemistry, Materials and Chemical Engineering "G. Natta", Politecnico di Milano, Via Bassini 6, 20133 Milano, Italy.

[2]ZHAW, Zurich University of Applied Sciences, IMPE, Institute of Materials and Processing Engineering, Technikumstrasse 9, 8401 Winterthur, Switzerland.

Corresponding author: Vincenzina Barbera (vincenzina.barbera@polimi.it)

† These authors equally contribute

Summary

S1. Materials and Methods 2
S2. Phosphorus-31 Nuclear Magnetic Resonance (^{31}P-NMR). 6
S3. Tollens' test 8
S4. Carbonyl groups content determination 9
S5. Hansen solubility parameters of LGN and LGN-CHO. Dispersion of LGN and LGN-CHO in different solvents 10
S6. FTIR-ATR 12
S7. Solvent resistance 13
S8. Resistance in water at different pH 16
S9. Regeneration of CS/LGN-CHO aerogels. 18
S10. Heavy metal removal using CS/LGN-CHO aerogels 19
S11. DFT calculations 24
References 32

Summary of figures

Figure S 1 31P-NMR spectrum of LGN 7
Figure S 2 Tollens' test results of LGN-CHO and LGN water suspensions 9
Figure S 3 FTIR-ATR spectra of CS (black line), CS aerogel 1 (orange line), and CS aerogel 2 (green line) 12
Figure S 4 Solvent resistance of CS/LGN-CHO 1:1 aerogel 13
Figure S 5 Solvent resistance of CS/LGN-CHO 1.5:1 aerogel 13
Figure S 6 Solvent resistance of CS/LGN-CHO 2:1 aerogel 14
Figure S 7 CS/LGN-CHO 1:1 aerogel in water at different pH 17
Figure S 8 CS/LGN-CHO 1.5:1 aerogel in water at different pH 17
Figure S 9 CS/LGN-CHO 2:1 aerogel in water at different pH 17
Figure S 10 A. FT-IR spectra of the acid-treated aerogel CS/LGN-CHO 1:1; A1. Regenerated aerogel; A2. Treated aerogel (pH 4, 30 days); A3. Untreated aerogel; A4. Chitosan; A5. Formylated Lignin; B. SEM micrograph of: untreated aerogel (B1); regenerated aerogel (B2); C. Catalytic performances of the regenerated aerogel. 19
Figure S 11 Filtration system adopted for heavy metals removal from contaminated water solution 20
Figure S 12 Representation of the HOMO, LUMO and Egap of: (A) CS/LGN-CHO; (B) optimized structures of the complex CS/LGN-CHO-Cu2+, (C) the complex CS/LGN-CHO-Zn2+, (D) the complex CS/LGN-CHO-Pb2+ 24

Summary of tables

Table S 1 Hydroxyl content of lignin determined through ^{31}P-NMR 8
Table S 2 Hansen solubility parameters for selected solvents and results of inspection of dispersions of LGN and LGN-CHO after 1 week[a,b] 10
Table S 3 Results of inspection of dispersions of LGN and LGN-CHO in various solvents[a,b]. 11
Table S 4 Weights of CS/LGN-CHO 1:1 aerogels. 14
Table S 5 Weights of CS/LGN-CHO 1.5:1 aerogel. 14
Table S 6 Weights of CS/LGN-CHO 2:1 aerogel. 14
Table S 7 Weights of CS/LGN-CHO 1:1 aerogel 17
Table S 8 Weights of CS/LGN-CHO 1.5:1 aerogel 17
Table S 9 Weights of CS/LGN-CHO 2:1 aerogel 17
Table S 10 Concentration of heavy metals detected using CS aerogel as adsorbent 20
Table S 11 Concentration of heavy metals detected using CS/LGN-CHO 1:1 aerogel as adsorbent 21
Table S 12 Concentration of heavy metals detected using CS/LGN-CHO 1.5:1 aerogel as adsorbent 21
Table S 13 Concentration of heavy metals detected using CS/LGN-CHO 2:1 aerogel as adsorbent 22
Table S 14 Results of the batch absorption test. 22
Table S 15 Second-order perturbation theory analysis of Fock matrix in NBO basis. NBO Interactions (E(2) > 1.0 kcal/mol) for Cu^{2+} complex 24
Table S 16 Second-order perturbation theory analysis of Fock matrix in NBO basis. NBO Interactions (E(2) > 1.0 kcal/mol) for Pb^{2+} complex 26
Table S 17 Second-order perturbation theory analysis of Fock matrix in NBO basis. NBO Interactions (E(2) > 1.0 kcal/mol) for Zn^{2+} complex 27
Table S18 Cartesian coordinates of all investigated structures (Angstroms) 29
Table S 19. Atomic charges (Hirshfeld population analysis) for ONO donor atoms and metal centers in CS/LGN-CHO complexes 31

S1. Materials and Methods

Materials

All reagents and solvents commercially available were used without further purification: lignin (LGN, obtained through Metso' LignoBoost method, iGreen), chitosan (CS, Mw: 110,000–150,000; degree of acetylation: ⩽40 mol. %), chloroform, acetic acid, pyridine, deuterated chloroform, N-hydroxy-5-norbornene-2,3-dicarboximide (NHND), 2-chloro-4,4,5,5-tetramethyl-1,3,2-dioxaphospholane (TMDP), chromium (III) acetylacetonate, hydroxylamine hydrochloride, triethanolamine (TEA), ethanol, dimethyl sulfoxide, acetone, 2-propanol, ethyl acetate, chloroform, xylene, hexane, dimethylformamide (Sigma-Aldrich), potassium hydroxide, silver nitrate, sodium hydroxide, aqueous ammonia, aqueous hydrochloric acid, sodium hydrogencarbonate, lead (II) nitrate, zinc chloride, copper (II) nitrate (Carlo Erba Reagenti).

Methods

Preparation of LGN-CHO by means of Reimer-Tiemann reaction performed on lignin

In a round-bottomed flask equipped with a magnetic stirrer and a condenser, potassium hydroxide (KOH, 3.12 g, 55 mmol), chloroform ($CHCl_3$, 1.12 mL, 14 mmol), and water (0.5 mL) were added sequentially. Lignin (0.500 g) was then introduced immediately to minimize the decomposition of chloroform by alkaline ions, a known issue in the Reimer–Tiemann reaction involving phenolic substrates. The resulting mixture was stirred at room temperature for 12 hours. After completion, the solvent was removed under reduced pressure. The obtained solid was finely ground in a mortar and pestle, transferred to a 15 mL Falcon™ tube, and dispersed in water (10 mL). The suspension

was sonicated for 10 minutes and centrifuged at 4000 rpm for 10 minutes; this washing step was repeated three times. After the formylation step, the LGN-CHO solid was purified by repeated cycles of dispersion in water/ethanol, centrifugation, and washing to remove soluble low-molecular-weight species and inorganic residues. The washing/centrifugation steps were repeated until no detectable low-molecular-weight residues remained in the washing steps, as confirmed by GC-MS analysis of the aqueous/alcohol washes, along with spectroscopic and thermal characterization of the purified solid (FTIR and TGA). Additionally, X-ray diffraction was used to exclude the presence of residual KCl, a common inorganic co-product of the Reimer-Tiemann reaction. The final product, designated as LGN-CHO, was obtained as a solid (0.7 g).

Preparation of the aerogels

Three samples with different mass ratios of chitosan (CS) and formylated lignin (LGN-CHO) were prepared: CS/LGN-CHO 1:1, CS/LGN-CHO 1.5:1 and CS/LGN-CHO 2:1. Chitosan and the formylated lignin were mixed for 5 min in a mortar with the help of a pestle. 50 mL of distilled water and 25 drops of an aqueous solution of acetic acid 99.7% (0.025g, 24 mmol) were added. The so obtained suspensions were sonicated for 30 min. The same procedure was deployed for the preparation of the reference sample based on pristine chitosan (CS).

The obtained water suspensions were frozen to −30°C, then lyophilized (EDWARDS MODULYO EF4-1596) under the following experimental conditions: T = −50°C, P = 5 mbar, lyophilization time t = 24 h. Four samples were obtained, consisting of an aerogel with only chitosan (CS aerogel) as a reference sample and three samples with different mass ratios of chitosan and formylated lignin (CS/LGN-CHO 1:1 aerogel, CS/LGN-CHO 1.5:1 aerogel, and CS/LGN-CHO 2:1 aerogel).

Preparation and filtration procedure of heavy metals contaminated solution using CS aerogel and CS/LGN-CHO aerogels.

Four aqueous solutions (1 L) containing Pb^{2+}, Cu^{2+} and Zn^{2+} as heavy metals were prepared. The final concentration of each metal was 15 mg L^{-1}. The required amount of starting materials ($Pb(NO_3)_2$ 24mg, $ZnCl_2$ 31mg, $Cu(NO_3)_2$ 44mg) was poured into a beaker and stirred for 1 h at room temperature. Each solution was filtered by using different aerogels (CS aerogel and CS/LGN-CHO aerogels) as the filter. The simultaneous removal of the abovementioned heavy metals was checked.

Regeneration of CS/LGN-CHO aerogels.

The regeneration of CS/LGN-CHO aerogels was performed by exploiting the pH sensitivity of the imine bonds. Following the conclusion of the adsorption or catalytic tests, the aerogel monoliths were immersed in an acidic aqueous solution (pH 4, adjusted with 0.1 M HCl) at room temperature. The samples were stirred for 24 hours, until complete network disassembly was observed. The application of this acidic treatment results in the cleavage of the imine bonds, consequently leading to the loss of the three-dimensional structure.

The resulting suspension was subsequently neutralised by gradual addition of a basic aqueous solution (0.1 M NaOH), thus restoring neutral pH and thereby promoting reformation of the imine-linked network. The regenerated material was collected, thoroughly washed with deionized water to remove residual salts, and finally freeze-dried to obtain regenerated aerogels as described previously.

The structural reversibility of the system was verified by FTIR-ATR spectroscopy, which demonstrated the reversible disappearance and reappearance of the imine (C=N) and carbonyl

(C=O) bands. Furthermore, the morphological features of the regenerated aerogels were examined by SEM. The regenerated aerogels were subsequently tested in catalytic cross-coupling reactions under the same conditions reported in the manuscript. The corresponding catalytic performance data are reported in Figure S10.

General Procedure for Chan-Lam Cross-Coupling Reactions

Imidazole (1.2 mmol), phenylboronic acid (1.0 mmol), Na_2CO_3 (1.0 mmol), and CS/LGN-CHO–Cu^{2+} aerogel (20 wt% with respect to phenylboronic acid) were dispersed in ethanol (4 mL), forming a heterogeneous suspension. The resulting reaction suspension was stirred at 60 °C for 24 h. The progress of the reaction was monitored by thin-layer chromatography (TLC). After completion, the mixture was allowed to cool to room temperature, and the solid aerogel catalyst was separated by filtration. The ethanolic filtrate was then concentrated under reduced pressure. The crude product was purified by silica gel column chromatography (cyclohexane/ethyl acetate) and characterized by ^{1}H NMR and ^{13}C NMR spectroscopy. Yellowish oil; ^{1}H NMR (400 MHz, $CDCl_3$) δ 7.86 (s, 1H), 7.45 (t, *J* = 7.8 Hz, 2H), 7.41–7.31 (m, 3H), 7.28 (s, 1H), 7.21 (s, 1H); ^{13}C NMR (100 MHz, $CDCl_3$) δ 137.3, 135.5, 130.2, 129.8, 127.5, 121.4, 118.1.

Reusability test

To assess catalyst durability and reusability, the CS/LGN-CHO–Cu^{2+} aerogel was recovered by filtration after each reaction cycle, thoroughly washed with ethanol to remove residual reactants or products, and subsequently reused in a new Chan–Lam cross-coupling reaction under identical conditions. Reaction yields were estimated by GC–MS analysis of the crude reaction mixtures.

Yields were estimated by GC-MS analysis on the crude product.

Theoretical Calculations

Molecular structures were initially built using Marvin Sketch (version 18.24, ChemAxon Ltd., Budapest, Hungary). Three-dimensional conformations were first generated using the Merck Molecular Force Field (MMFF94), followed by semiempirical optimization employing the parameterized model number 6 (PM6) Hamiltonian, as implemented in the MOPAC package (MOPAC2016 v. 18.151, Stewart Computational Chemistry, Colorado Springs, CO, USA) [1,2] , prior to performing DFT calculations.

The DFT calculations were performed using the Gaussian 09 software (Gaussian, Inc., Wallingford, CT, USA) with B3LYP and LANL2DZ basis sets[3]. The LANL2DZ basis set is well-suited for describing metal–ligand interactions and is considered computationally efficient[4]. The 3D structures and molecular electrostatic potential surfaces (MEPS) were obtained using the Spartan'24 package.[5]

Characterizations

^{31}Phosphorus Nuclear Magnetic Resonance (^{31}P-NMR).

^{31}P-NMR spectra were recorded by using a Bruker AV 400 (162 MHz). Phosphorylation of lignin followed by ^{31}P-NMR analysis is a good approach to characterize the hydroxyl groups in a material such as lignin. The selected phosphorylating agent, TMDP, reacts with the different types of OH groups. Aliphatic, phenolic, and carboxylic OH may be readily quantified with ^{31}P-NMR spectroscopy. The analysis was conducted to check the amount of aliphatic hydroxyl groups of lignin, which were then used to determine the minimum amount of reagents (KOH and $CHCl_3$) for the Reimer-Tiemann reaction required (Figure S1, Table S1).

Tollens' test.

Tollens' reagent was prepared by mixing together 50 mL of aqueous silver nitrate (0.1 M) and 50 mL of aqueous sodium hydroxide (0.8 M). Aqueous ammonia was added dropwise until the precipitated silver oxide completely dissolved. Once Tollens' reagent was ready, 0.5 g of LGN and LGN-CHO were placed into two different glass test tubes, and 10 mL of Tollens' reagent was added in each tube. The tubes were sonicated for 15 min in order to facilitate the solubilization of LGN and LGN-CHO, respectively. The tubes were then heated at 50 °C for 1 h, then stored at room temperature for 24 h.

Carbonyl group content determination.

The carbonyl group content quantification was carried out on lignin (LGN) and on formylated lignin (LGN-CHO) by oximation. Oximating solution was prepared dissolving 1.2 g of TEA in 96% alcohol in a 50 ml volumetric flask (TEA stock). In a second 50 ml volumetric flask, 0.7 g of NH_2OH*HCl was dissolved in a 5 ml of deionized water. From TEA stock, 25 ml solution was taken and put into the second flask. For carbonyl determination, 80 mg of sample was placed into a sealable cap-tube and dissolved in 2 mL DMSO. 5 mL of oximating solution was added. Air in the tube was expelled with nitrogen and sealed. The closed tube was stirred and heated at 80 °C for 2 hours. The cool solution was then quantitatively transferred into a beaker, and a little amount of water (about 1 mL) was added. The excess of TEA was potentiometrically titrated with 0.1 N HCl to pH 3.3.

Attenuated Total Reflection Fourier Transform Infrared spectroscopy (FTIR-ATR).

The FTIR-ATR spectra were recorded on LGN, LGN-CHO, CS, CS aerogel, CS/LGN-CHO physical mixture, and CS/LGN-CHO aerogels. All spectra were recorded in absorbance mode (64 scans and 4 cm^{-1} resolution) using a Thermo Scientific Nicolet iS5 FT-IR Spectrometer, with a diamond crystal fixed at the incident angle of 45°. A little amount of lignin was placed on the top of the crystal and pressed gently by a pre-mounted sample clamp.

Thermogravimetric Analysis (TGA).

Thermogravimetric analyses were conducted on LGN, LGN-CHO, CS, CS aerogel, and CS/LGN-CHO aerogels. The tests were conducted deploying a TA Instruments Q5000 IR and a TGA/DSC 3+ (Mettler Toledo), with a temperature ramp of 10 °C/min from 25 °C to 900 °C under a nitrogen flow of 25 cm^3 min^{-1} atmosphere. About 10 mg of all samples were used for the analysis.

Hansen solubility parameters determination (HSP).

HSP were determined for LGN and LGN-CHO. Dispersions of 1 mg/mL were prepared in water, methanol, acetone, 2-propanol, ethyl acetate, chloroform, xylene, and hexane, respectively. Mixtures were sonicated for 15 minutes. For each dispersion the stability was evaluated as a function of time: (i) at rest, (ii) 1 hour, (iii) 1 week.

Scanning electron microscopy (SEM).

The morphologies of CS/LGN-CHO aerogels were analyzed by scanning electron microscopy (SEM). The specimens, mounted onto aluminum stubs, were sputter-coated with gold and examined with a StereoScan 360 Cambridge microscope at 10 kV. Samples were observed at 2000× and 10000× magnification, respectively.

Solvent resistance.

Tests were performed placing CS/LGN-CHO aerogels (aerogels: 1 cm, 3 mm thickness) in vials containing 2 mL of water, hexane, and dimethyl formamide (DMF), respectively. Vials were left for 48h to test the solvent stability. The stability of the material was then measured by means of weight loss.

pH resistance.

Tests were performed placing CS/LGN-CHO aerogels (aerogels: 1 cm^2, 3 mm thickness) in vials containing 2 mL of water solutions at different pH. Vials were left for 1 month. Acids and bases used to tune pH were: HCl (38%), CH_3COOH (99.85%), $NaHCO_3$, KOH. The stability of the material was then measured by means of weight loss.

Inductively coupled plasma atomic emission spectroscopy (ICP-OES).

Analysis was conducted on samples collected following the filtration of the heavy metal contaminated solution through CS aerogel and CS/LGN-CHO aerogels. The concentrations of the heavy metals (Pb^{2+}, Cu^{2+} and Zn^{2+}) were detected using an ICP-OES Perkin-Elmer Precisely - OPTIMA 2100DV. Samples of 3 mL were collected after the following volumes of filtered contaminated solution: 50 mL, 100 mL, 150 mL, 200 mL, 300 mL, 500 mL.

X-Rays Photoelectron Spectroscopy (XPS).

Survey and high-resolution XPS spectra were collected using a PHI 5000 VersaProbe instrument (Physical Electronics). Measurements were performed under ultra-high vacuum (UHV) conditions (≈10^{-8} Pa) with a monochromatic Al Kα X-ray source (1486.6 eV), operated at 15 kV and 1 mA (25.2 W). Survey and high-resolution spectra were acquired with pass energies of 187.85 eV and 23.5 eV, respectively. Analyses were conducted at a take-off angle of 45° using a 100 µm X-ray spot and a dual-beam charge neutralization system (electron and Ar^+ ion gun) to minimize surface charging. Samples were stored overnight in the XPS pre-chamber prior to analysis to prevent anomalous outgassing. Spectra were processed using MultiPak 9.6 software, with binding energies referenced to the C 1s peak at 284.5 eV. Peak fitting of the high-resolution Cu 2p spectra was performed using PeakFit software.

Statistical analysis

Dynamic Adsorption: Breakthrough Analysis experiments were performed in triplicate (n=3). Error bars in graphical representations indicate the range between the minimum and maximum values recorded for each data point. Catalytic cycle analysis was performed in triplicate (n=3) and the error bars represents SD. For the determination of pore size via SEM, 10 independent measurements were performed for each sample. Data are presented as mean ± standard deviation (SD). Statistical significance was assessed using One-way ANOVA. A P value < 0.05 was considered statistically significant. All statistical analyses and regressions were performed using OriginPro 2018.

S2. Phosphorus-31 Nuclear Magnetic Resonance (^{31}P-NMR).

^{31}P-NMR was performed on lignin (LGN) using a Bruker AV 400 (162 MHz). The analysis was to determine the hydroxyl content of lignin[6], useful for determining the amount of reagents (KOH, $CHCl_3$) to be used in the Reimer-Tiemann reaction for the synthesis of formylated lignin (LGN-CHO).

Sample preparation.

A solvent mixture composed of pyridine and chloroform-d1 in a 1.6:1 v/v ratio was prepared. 40 mg of NHND and 40 mg of chromium (III) acetylacetonate were dissolved in 10 mL of the above preparation to obtain a solution with an internal standard and relaxation reagent, respectively. 40 mg of dry lignin and 0.1 mL internal standard/relaxation solution were added into 0.8 mL pyridine/chloroform-d1 (1.6:1 v/v) solvent, and the mixture was stirred at room temperature until the lignin dissolved. Finally, 130 µL of TMDP was added and the mixture was transferred into a 5 mm NMR tube. **Figure S1** show the obtained spectra.

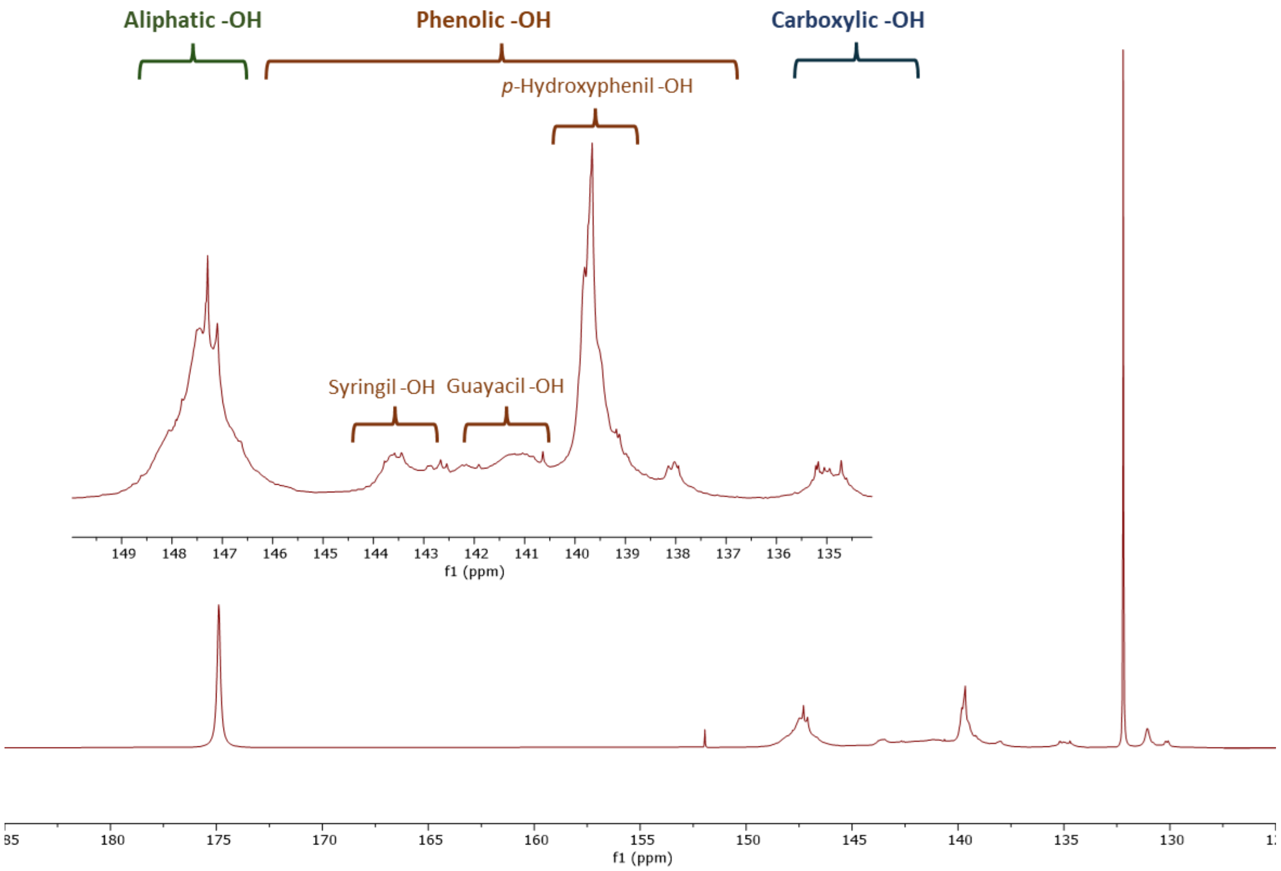

Figure S 1 *^{31}P-NMR spectrum of LGN*

Calculations for hydroxyl content determination.

The following equations were used in sequence:

- Calculation of the mmol of the Internal Standard (NHND, 97% purity) in Internal Standard solution (IS) (Eq. 1):

$$\text{mmol NHND in IS} = \frac{\text{Mass of NHND (g)}}{\text{MW NHND}} \text{ x } 97\% \text{ x } 1{,}000 \qquad \textbf{Eq. 1}$$

- Calculation of mmol NHND in NMR sample (Eq. 2):

$$\text{mmol NHND in NMR sample} = \frac{\text{NHND in IS solution (mmol)}}{\text{Total mass of IS solution (g)}} * \text{Mass of 0,1 ml of IS (g)} \qquad \textbf{Eq. 2}$$

- Calculation of the ratio (R) of the integration of the spectral region of interest (I_{OH}) over the IS region (I_{NHND}) (Eq. 3):

$$R = \frac{IOH}{INHND} = \frac{\text{Integration of spectral region of interest}}{\text{Integration of NHND region}}$$ **Eq.3**

- Calculation of the amount of different hydroxyls groups (Eq. 4):

$$\text{mmol OH/g}_{\text{lignin}} = \frac{\text{R x mmol of NHND in NMR sample (mmol)}}{\text{Dry weight of lignin (g)}}$$ **Eq. 4**

The results obtained using this procedure and equations are in Table S1.

Table S 1 *Hydroxyl content of lignin determined through ^{31}P-NMR*

**Structure**	mmol OH/g $_{\text{lignin}}$
**Aliphatic OH**	3.62
**Syringyl OH**	0.57
**Guaiacyl OH**	1.36
**p-Hydroxyphenyl OH**	0.68
**Carboxylic OH**	0.26

For the Reimer-Tiemann reaction carried out on LGN, aromatic hydroxyls (Syringyl OH, Guaiacyl OH and p-hydroxyphenyl OH) were considered. Therefore, 2.61 mmol of OH were considered to determine the KOH and $CHCl_3$ required for the formylation reaction.

S3. Tollens' test

Tollens' test[7] was conducted on lignin (LGN) and on formylated lignin (LGN-CHO) to check the presence of aldehydic groups after the Reimer-Tiemann reaction.

Preparation of the Tollens' reagent

The Tollens test involves the use of a colorless, basic aqueous solution containing silver ions coordinated with ammonia $[Ag(NH_3)^{2+}]$. This solution was prepared using a two-step procedure:

- STEP 1. 50 mL of aqueous silver nitrate (0.1 M) was mixed with 50 mL of aqueous sodium hydroxide (0.8 M) to lead to the formation of silver hydroxide, which in turn dissociates to give silver oxide:

$$AgNO_3 + NaOH \rightarrow AgOH + NaHO_3$$
$$2\,AgOH \rightarrow Ag_2O + H_2O$$

- STEP 2. Aqueous ammonia was added dropwise until the precipitated silver oxide completely dissolved. $Ag(NH_3)^{2+}$ was obtained in the solution:

$$Ag_2O + 4\,NH_3 + H_2O \rightarrow 2\,Ag(NH_3)_2^+ + 2\,OH^-$$

Experimental procedure

0.5 g of LGN (or LGN-CHO) was placed in a glass test tube, and 10 mL of Tollens' reagent was added. The mixture was sonicated for 15 min in order to facilitate the solubilization of LGN (or LGN-CHO). The mixture was then heated at 50 °C for 1 h, then stored at room temperature for 24 h.

The results of this qualitative test are shown in the following **Figure S2**.

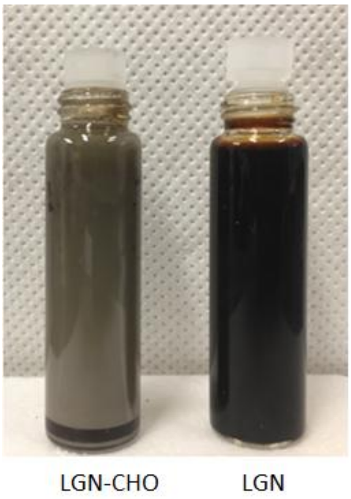

Figure S 2 *Tollens' test results of LGN-CHO and LGN water suspensions*

S4. Carbonyl groups content determination

The content of carbonyl groups was determined on lignin (LGN) and formylated lignin (LGN-CHO) by oximation[8].

Preparation of oximating solution

1.2 g of TEA was dissolved in 96% alcohol in a 50 ml volumetric flask (TEA stock). In a second 50 ml volumetric flask, 0.7 g of NH_2OH*HCl was dissolved in a 5 ml of deionized water. From TEA stock, 25 ml solution was taken and put into the second flask. The second volumetric flask was brought to volume (50 ml) with 96% ethanol.

Procedure for carbonyl group determination.

80 mg of sample was placed into a sealable cap tube and dissolved in 2 mL of DMSO. 5 mL of oximating solution was added. Air in the tube was expelled with nitrogen and sealed. The closed tube was stirred and heated at 80 °C for 2 hours. The cool solution was then quantitatively transferred into a beaker, and a little amount of water (about 1 mL) was added. The excess of TEA was potentiometrically titrated with 0.1 N HCl to pH 3.3. A blank experiment without sample, but under the same condition as before, was performed using the oximating solution (blank a). Two additional blank experiments were necessary: blank b (experiment with TEA stock solution + 2 ml DMSO in the absence of sample) and blank c (experiment with TEA stock solution + 2 ml DMSO in the presence of sample).

Blank b and blank c are required because the TEA used is in excess, and carboxylic or other strong acidic groups in lignin may consume TEA during oximation. This is a source of apparent CO increase. To avoid errors, a second pair of blanks is necessary.

Calculation for carbonyl content.

Eq. 5 was used for the quantitative determination of carbonyl groups (expressed as % of the amount of sample analyzed).

$$\%CO = \left(\frac{a_0 - a}{A} - \frac{b_0 - c_0}{C}\right) * f * 280.1 \qquad \textbf{Eq. 5}$$

where: a_0 = volume of 0.1 N HCl (ml) used for blank titration; a = volume of 0.1 N HCl (ml) used for sample titration; b_0 = volume of 0.1 N HCl (ml) used for blank b titration; c_0 = volume of 0.1 N HCl (ml) used for blank c titration; A = weight of lignin (mg) used for the analysis; C = weight of lignin (mg) in blank c; f = titer of 0.1 N HCl; 280.1 = mass of CO group (mg), equivalent to 1 ml of 0.1 N HCl multiplied by 100.

S5. Hansen solubility parameters of LGN and LGN-CHO. Dispersion of LGN and LGN-CHO in different solvents

The test was conducted on LGN and LGN-CHO. The samples were put in different solvents to investigate their solubility parameters.

Experimental procedure

Dispersions of 1 mg/mL were prepared in water, methanol, acetone, 2-propanol, ethyl acetate, chloroform, xylene, and hexane. Mixtures were sonicated for 15 minutes. For each dispersion, the stability as a function of time is (i) at rest, (ii) 1 hour, and (iii) 1 week. The results of the inspections are summarized in Table S2, where the label 'good' indicates that a homogenous dispersion was observed immediately after sonication and also after one week, while the label 'bad' indicates the separation of the adduct from the solvent.

Table S 2 *Hansen solubility parameters for selected solvents and results of inspection of dispersions of LGN and LGN-CHO after 1 week*[a,b]

**Solvent**	**δ_D [MPa$^{1/2}$]**	**δ_P [MPa$^{1/2}$]**	**δ_H [MPa$^{1/2}$]**	**Stability of dispersions**	
				**LGN**	**LGN-CHO**
water	18.1	17.1	16.9	bad	good
methanol	15.1	12.3	22.3	bad	good
2-propanol	15.8	6.1	16.4	bad	good
acetone	15.5	10.4	7.0	good	good
ethyl acetate	15.8	5.3	7.2	bad	bad
chloroform	17.8	3.1	5.7	bad	bad

xylene	17.6	1.0	3.1	bad	bad
hexane	14.9	0.0	0.0	bad	bad

[a] concentration: 1mg/mL; [b]good: homogenous dispersion was observed soon after sonication and after one week storage at rest; bad: separation of adduct from the solvent

The qualitative "good" or "bad" classification of Table S2 was used to identify the unknown Hansen Solubility Parameters of LGN and LGN-CHO, by applying a fitting sphere algorithm as explained in the next section. In Table S3 are summed up the solvents used with their Hansen solubility parameters (HSP), together with the affinity toward LGN and LGN-CHO after one-week storage at rest.

Table S 3 *Results of inspection of dispersions of LGN and LGN-CHO in various solvents[a,b].*

Sample	***solvents***							
	water	methanol	acetone	2-propanol	ethyl acetate	chloroform	xylene	hexane
**LGN**	bad	bad	good	bad	bad	bad	bad	bad
**LGN-CHO**	good	good	good	good	bad	bad	bad	bad

[a] concentration: 1mg/mL; [b]good: homogenous dispersion was observed soon after sonication and after one week storage at rest; bad: separation of adduct from the solvent.

Calculation of the Hansen Solubility Sphere and Hansen Solubility Parameters

The calculation of the Hansen Solubility Parameters (HSP) for LGN and LGN-CHO samples was performed applying the Hansen Solubility Sphere representation of miscibility. The idea at the basis of this geometrical approach is the calculation of the cohesive energy density (U_T/V) of a compound as the sum of three interaction contributions, as given by Equation 6: dispersion (non polar Van der Waals forces) (δ_D), polar (δ_P) and hydrogen bonding (δ_H).

$$\frac{U_T}{V} = \delta_T^2 = \delta_D^2 + \delta_P^2 + \delta_H^2 \quad \textbf{Eq. 6}$$

The compound is therefore identified by three coordinates (δ_D, δ_P, and δ_H) in the Hansen Parameters space. The distance between two points (e.g. a solute and its solvent) is related to their cohesive energy density difference, which is related to the enthalpy of mixing. As the enthalpy of mixing is minimal for miscible substances, two points close to each other in the Hansen space correspond to miscible compounds.

To estimate the HSP of a solute i, a dispersion test is performed on different solvents j, distinguishing good solvents (providing stable solutions/dispersions) and bad solvents, which are not able to give stable dispersions. Given the parameters (coordinates) of the solvents, it is possible to define a sphere, centered on the solubility parameters of the solute, which encompasses the good solvents and excludes the non-solvents. The sphere radius is defined as R_0, the radius of interaction, while the distance between the solute and the solvent is R_a, calculated as in Equation 7.

$$R_{a,ij}^{\ 2} = 4(\delta_{D,i} - \delta_{D,j})^2 + (\delta_{P,i} - \delta_{P,j})^2 + (\delta_{H,i} - \delta_{H,j})^2 \quad \textbf{Eq. 7}$$

The ratio between $R_{a,ij}$ and R_0 is defined in Equation 8 as RED, relative energy difference. Solutes and solvents with good affinity have a relative energy difference lower than 1.

$$RED = \frac{R_{a,ij}}{R_o} \quad \textbf{Eq. 8}$$

An optimization problem is therefore defined: the center coordinates of the Hansen solubility sphere are calculated by minimizing the radius of interaction (i.e. the distance from the coordinates of the good solvents), including the good solvents (RED < 1) and excluding the bad ones. The sphere center coordinates correspond to the three unknown HSP of the solute.

The fitting sphere program was adapted from[9] and solved in Matlab environment using the Nelder-Mead simplex algorithm.

A more detailed explanation of the theoretical basis of the Hansen solubility parameters as well as of the algorithm used to estimate the parameters' values has been reported elsewhere[10].

S6. FTIR-ATR

In Figure S3 are reported the FTIR-ATR spectra of chitosan powder (CS), chitosan aerogel 1 (CS aerogel), and chitosan aerogel 2 (CS aerogel).

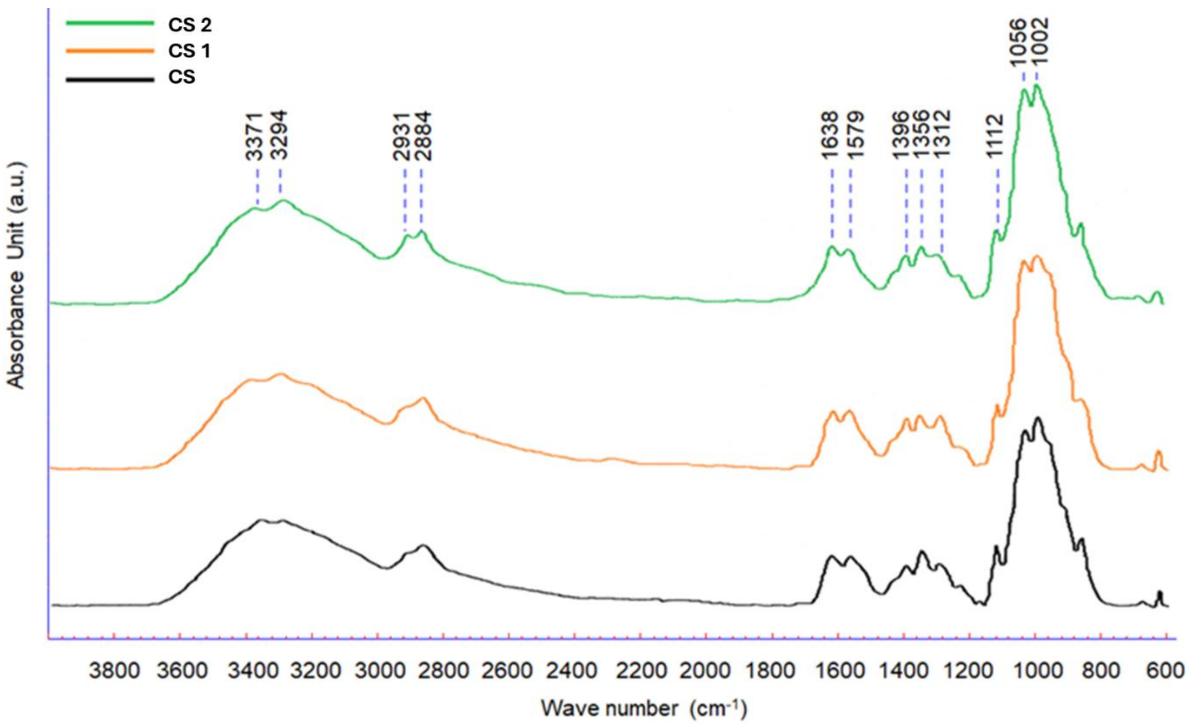

Figure S 3 FTIR-ATR spectra of CS (black line), CS aerogel 1 (orange line), and CS aerogel 2 (green line)

All the spectra show the same profile. A strong band in the region 3371-3294 cm^{-1} corresponds to N-H and O-H stretching, as well as the intramolecular hydrogen bonds. The absorption bands at around 2931 and 2884 cm^{-1} can be attributed to C-H symmetric and asymmetric stretching, respectively. The presence of residual N-acetyl groups was confirmed by the bands at around 1638 cm^{-1} (C=O stretching of primary amide) and 1312 cm^{-1} (C-N stretching of tertiary amide). A band at 1579 cm^{-1} corresponds to the N-H bending of the primary amine. The CH_2 bending and CH_3 symmetrical deformations were confirmed by the presence of bands at around 1396 and 1356 cm^{-1}, respectively. The absorption peak at 1112 cm^{-1} can be attributed to asymmetric stretching of the C-O-C bridge. The bands at 1056 and 1002 cm^{-1} correspond to C-O stretching.

S7. Solvent resistance

Solvent resistance of CS/LGN-CHO aerogels was checked by putting samples in vials containing different solvents (water, n-hexane, DMF). Figures S4, S5, and S6 show the vials containing CS/LGN-CHO composites, while Tables S4, S5, and S6 show the mass of all samples before and after the storage.

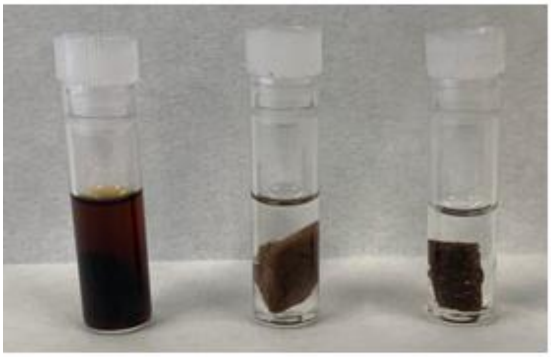

DMF water hexane

Figure S 4 Solvent resistance of CS/LGN-CHO 1:1 aerogel

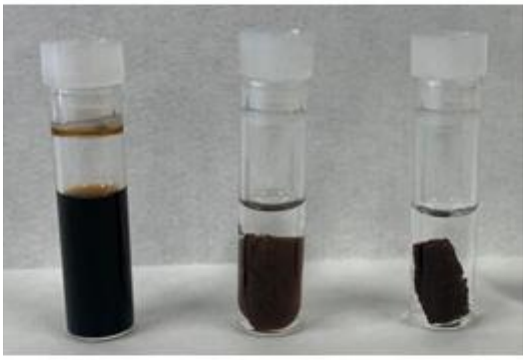

DMF water hexane

Figure S 5 Solvent resistance of CS/LGN-CHO 1.5:1 aerogel

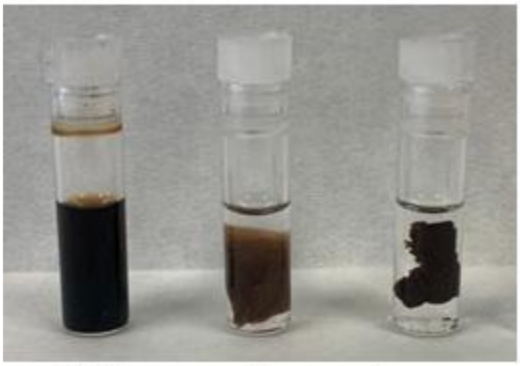

Figure S 6 *Solvent resistance of CS/LGN-CHO 2:1 aerogel*

Table S 4 *Weights of CS/LGN-CHO 1:1 aerogels.*

**Sample**	**Solvents**[a]		
	DMF	H_2O	Hexane
CS/LGN-CHO aerogel[b]	0.00793 g	0.00375 g	0.00470 g
CS/LGN-CHO aerogel[c]	n.d.[d]	0.01275 g	0.00475 g

[a] 2.5 mL; [b] grams of sample before; [c] grams of sample after 48h; [d] not determined = swelling and breakup of speciments.

Table S 5 *Weights of CS/LGN-CHO 1.5:1 aerogel.*

**Sample**	**Solvents**[a]		
	DMF	H_2O	Hexane
CS/LGN-CHO aerogel[b]	0.00718 g	0.00721 g	0.00706 g
CS/LGN-CHO aerogel[c]	n.d.[d]	0.01321 g	0.00723 g

[a] 2.5 mL; [b] grams of sample before; [c] grams of sample after 48h; [d] not determined = swelling and breakup of speciments.

Table S 6 *Weights of CS/LGN-CHO 2:1 aerogel.*

**Sample**	**Solvents**[a]		
	DMF	H_2O	Hexane
CS/LGN-CHO aerogel[b]	0.00369 g	0.00403 g	0.00291 g
CS/LGN-CHO aerogel[c]	n.d.[d]	0.01225 g	0.00312 g

[a] 2.5 mL; [b] grams of sample before; [c] grams of sample after 48h; [d] not determined = swelling and breakup of speciments.

S8. Resistance in water at different pH

The pH resistance of CS/LGN-CHO aerogels in water at different pH was investigated by using HCl, CH_3COOH, $NaHCO_3$ and KOH to modulate the pH. Figures S7 to S9 show the vials containing CS/LGN-CHO composites, while Tables S7 to S9 show the mass of all samples before and after the storage.

pH	Picture	Time to disruption (days)
1	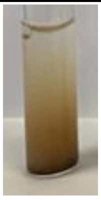	5
4	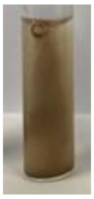	7
5	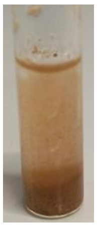	9
6	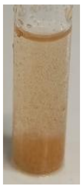	14
7	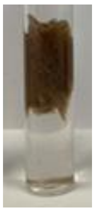	nd[a]
9	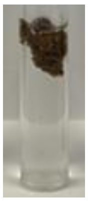	nd[a]
10	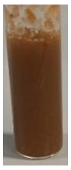	20

pH	Picture	Time to disruption (days)
1	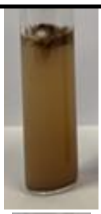	6
4	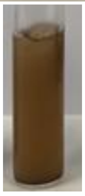	8
5	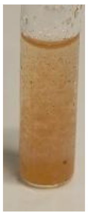	11
6	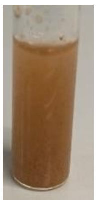	17
7	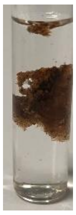	nd[a]
9	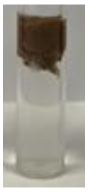	nd[a]
10	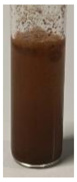	24

pH	Picture	Time to disruption (days)
1	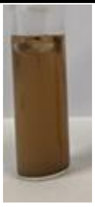	8
4	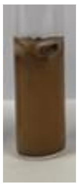	12
5	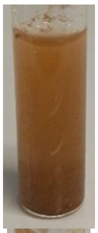	18
6	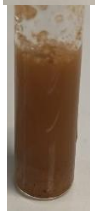	22
7	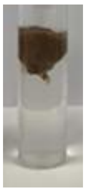	nd[a]
9	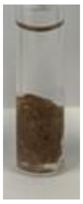	nd[a]
10	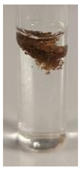	nd[a]

14	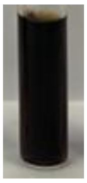	17	14	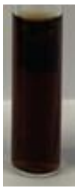	20	14	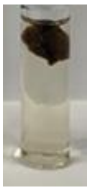	nd[a]

Figure S 7 *CS/LGN-CHO 1:1 aerogel in water at different pH*

Figure S 8 *CS/LGN-CHO 1.5:1 aerogel in water at different pH*

Figure S 9 *CS/LGN-CHO 2:1 aerogel in water at different pH*

[a]nd: not determined

Table S 7 *Weights of CS/LGN-CHO 1:1 aerogel*

Sample	pH[a, b]				
	1	4	7	9	14
CS/LGN-CHO aerogel[c]	0.00221 g	0.00259 g	0.00340 g	0.00095 g	0.00143 g
CS/LGN-CHO aerogel[d]	n.d.[e]	n.d.[e]	0.00369 g	0.00096 g	n.d.[e]

[a] 2.5 mL; [b] water solutions; [c] grams of sample before; [d] grams of sample after 48h; [e] not determined = swelling and breakup of speciments.

Table S 8 *Weights of CS/LGN-CHO 1.5:1 aerogel*

Sample	pH[a, b]				
	1	4	7	9	14
CS/LGN-CHO aerogel[c]	0.00231 g	0.00255 g	0.00330 g	0.00086 g	0.00133 g
CS/LGN-CHO aerogel[d]	n.d.[e]	n.d.[e]	0.00369 g	0.00094 g	n.d.[e]

[a] 2.5 mL; [b] water solutions; [c] grams of sample before; [d] grams of sample after 48h; [e] not determined = swelling and breakup of speciments.

Table S 9 *Weights of CS/LGN-CHO 2:1 aerogel*

Sample	pH[a, b]

	1	4	7	9	14
CS/LGN-CHO aerogel[c]	0.00231 g	0.00255 g	0.00330 g	0.00086 g	0.00133 g
CS/LGN-CHO aerogel[d]	n.d.[e]	n.d.[e]	0.00369 g	0.00094 g	0.00176 g

[a] 2.5 mL; [b] water solutions; [c] grams of sample before; [d] grams of sample after 48h; [e] not determined = swelling and breakup of speciments.

S9. Regeneration of CS/LGN-CHO aerogels.

The regeneration of CS/LGN-CHO aerogels was performed by exploiting the pH sensitivity of the imine bonds. Following the conclusion of the adsorption or catalytic tests, the aerogel monoliths were immersed in an acidic aqueous solution (pH 4, adjusted with 0.1 M HCl) at room temperature. The samples were subjected to agitation for a period of seven days. This was done until such a time as complete network disassembly was observed. The resulting suspension was subsequently neutralised by gradual addition of a basic aqueous solution (0.1 M NaOH), thus restoring neutral pH and thereby promoting reformation of the imine-linked network. The regenerated material was collected, thoroughly washed with deionized water to remove residual salts, and finally freeze-dried to obtain regenerated aerogels as described previously. The structural reversibility of the system was verified by FTIR-ATR spectroscopy, as shown in figure S10 A, which demonstrated the reversible disappearance and reappearance of the imine (C=N) and carbonyl (C=O) bands. Furthermore, the morphological features of the regenerated aerogels were examined by SEM, reported in figure S10 B. The regenerated aerogels were subsequently tested in catalytic cross-coupling Chan-Lam reactions under the same conditions reported in the manuscript. The corresponding catalytic performance data are reported in Figure S10 C.

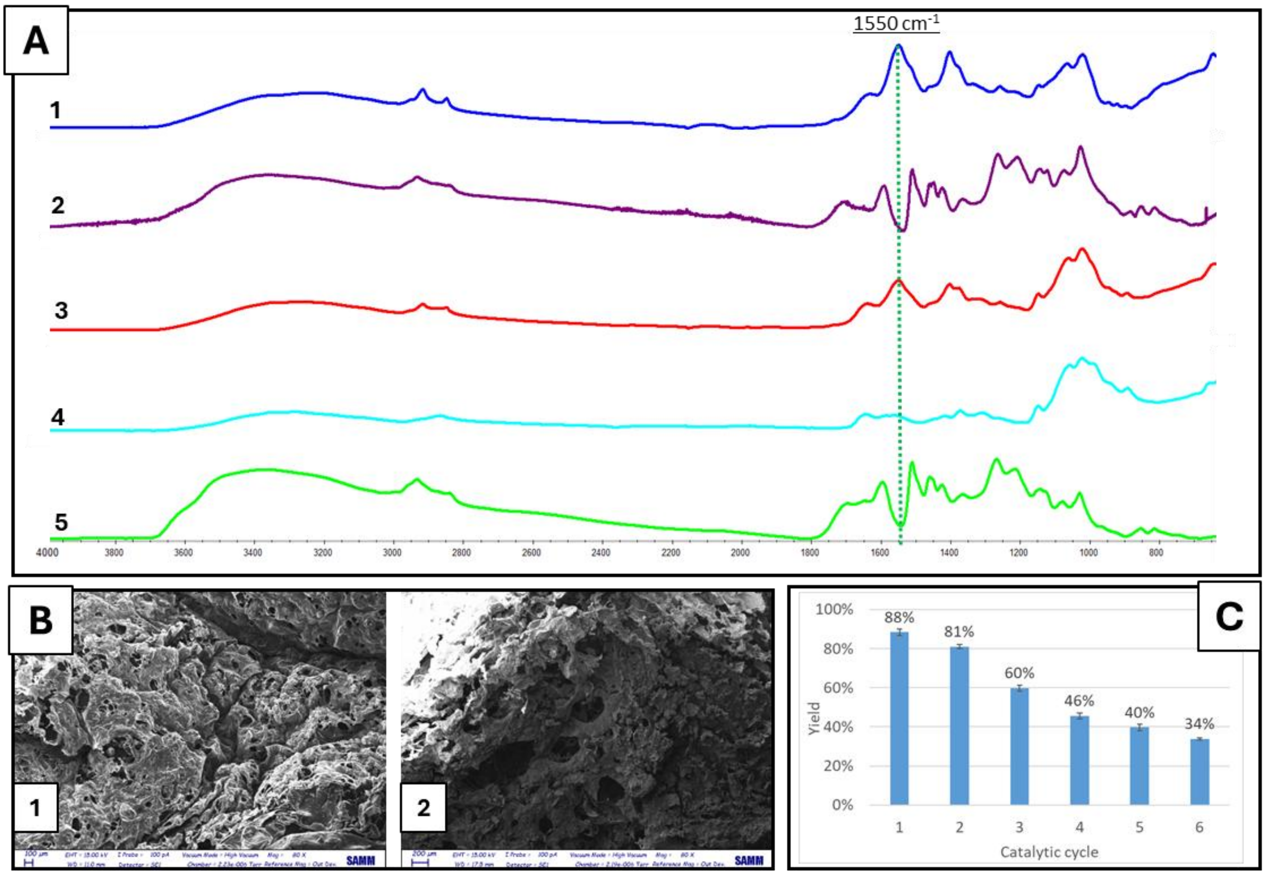

Figure S 10 A. *FT-IR spectra of the acid-treated aerogel CS/LGN-CHO 1:1;* ***A1****. Regenerated aerogel;* ***A2****. Treated aerogel (pH 4, 30 days);* ***A3****. Untreated aerogel;* ***A4****. Chitosan;* ***A5****. Formylated Lignin;* ***B****. SEM micrograph of: untreated aerogel (****B1****); regenerated aerogel (****B2****);* ***C****. Catalytic performances of the regenerated aerogel. Error bars represent the standard deviation (SD) calculated from three independent experimental replicates.*

S10. Heavy metal removal using CS/LGN-CHO aerogels

The adsorbent capacity of CS/LGN-CHO aerogels was tested for the simultaneous removal of heavy metals from contaminated water solutions. Each test was conducted using 2 g of composite. Solutions containing Pb^{2+}, Cu^{2+}, and Zn^{2+} as heavy metals were prepared and filtered.

Filtration procedure

The schematic representation of the filtration system is reported in Figure S11.

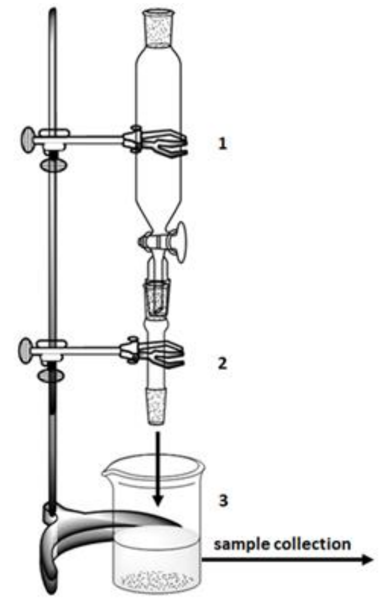

Figure S 10 *Filtration system adopted for heavy metals removal from contaminated water solution*

The heavy metal water solutions were put in a 1 L column equipped with a valve on the base (1, Figure S11). The solutions were dripped with a flow rate of 6 ml min^{-1} into a tube containing the testing aerogel (2, Figure S11). Samples of 3 mL were collected from the collection beaker (3, Figure S11) after the following volumes of filtered contaminated solution: 50 mL, 100 mL, 150 mL, 200 mL, 300 mL, and 500 mL.

ICP-OES

ICP-OES measurements were registered on collected samples to evaluate the concentration of heavy metals. Tables S11 to S14 report the registered metal concentrations. From the data reported in Tables S10 to S13, saturation kinetic-type curves were obtained as heavy metals removal % vs volume of treated solution. Heavy metal removal % values were obtained using Equation 9.

$$\text{Heavy metals removal \%} = 100\% - \left(\frac{100*ppm_i}{ppm_{starting}}\right)\% \qquad \textbf{Eq. 9}$$

where ppm_i is the concentration of heavy metals detected from ICP-OES in every analyzed sample and $ppm_{starting}$ is the initial metal concentration present in the solution (15 mg L^{-1} for every metal).

Table S 10 *Concentration of heavy metals detected using CS aerogel as adsorbent*

**Sample**	**Metals**		
	Pb^{2+}	Zn^{2+}	Cu^{2+}

	(mg L^{-1})			(mg L^{-1})			(mg L^{-1})		
CS_{50}[a]	7.71	7.79	7.65	8.3	8.33	8.12	9.11	9.05	8.99
CS_{100}[b]	13.24	13.31	13.19	12.61	12.70	12.43	13.42	13.38	13.30
CS_{150}[c]	14.54	14.50	14.34	14.58	14.49	14.46	14.62	14.60	14.46
CS_{200}[d]	14.12	14.55	14.17	14.31	14.56	14.11	14.73	14.70	14.55
CS_{300}[e]	14.71	14.81	14.68	14.73	14.66	14.70	13.69	14.35	13.55
CS_{500}[f]	14.63	14.80	14.59	14.81	14.92	14.85	14.24	14.30	14.14

[a, b, c, d, e, f] sample analyzed after 50, 100, 150, 200, 300, and 500 mL of filtered solution, respectively

Table S 11 *Concentration of heavy metals detected using CS/LGN-CHO 1:1 aerogel as adsorbent*

	**Metals**								
**Sample**	Pb^{2+} (mg L^{-1})			Zn^{2+} (mg L^{-1})			Cu^{2+} (mg L^{-1})		
CS/LGN_{50}[a]	4.21	4.24	4.16	4.71	4.66	4.57	5.90	5.94	5.60
CS/LGN_{100}[b]	5.16	5.20	5.11	4.94	5.07	4.95	6.34	6.44	6.20
CS/LGN_{150}[c]	9.12	9.13	9.09	10.30	10.13	10.25	12.64	12.72	12.55
CS/LGN_{200}[d]	13.44	13.29	13.40	14.65	14.73	14.71	14.32	14.41	14.25
CS/LGN_{300}[e]	14.71	14.64	14.59	14.13	14.35	14.22	14.84	14.90	14.70
CS/LGN_{500}[f]	14.74	14.71	14.63	14.78	14.80	14.71	14.16	14.22	14.10

[a, b, c, d, e, f] sample analyzed after 50, 100, 150, 200, 300, and 500 mL of filtered solution, respectively

Table S 12 *Concentration of heavy metals detected using CS/LGN-CHO 1.5:1 aerogel as adsorbent*

	**Metals**								
**Sample**	Pb^{2+} (mg L^{-1})			Zn^{2+} (mg L^{-1})			Cu^{2+} (mg L^{-1})		
CS/LGN_{50}[a]	3.42	3.54	3.38	3.71	3.77	3.68	4.65	4.72	4.57
CS/LGN_{100}[b]	4.23	4.32	4.12	3.86	4.01	3.73	5.74	5.82	5.70

CS/LGN$_{150}$^{c}	5.92	6.07	5.87	4.12	4.22	4.02	7.63	7.71	7.54
CS/LGN$_{200}$^{d}	12.64	12.81	12.58	13.76	13.81	13.68	13.87	14.01	13.65
CS/LGN$_{300}$^{e}	14.56	14.66	14.52	14.38	14.56	14.33	14.23	14.38	14.11
CS/LGN$_{500}$^{f}	14.74	14.81	14.67	14.67	14.76	14.64	14.57	14.71	14.66

[a, b, c, d, e, f] sample analyzed after 50, 100, 150, 200, 300, and 500 mL of filtered solution, respectively

Table S 13 *Concentration of heavy metals detected using CS/LGN-CHO 2:1 aerogel as adsorbent*

Sample	Metals								
	Pb^{2+} (mg L^{-1})			Zn^{2+} (mg L^{-1})			Cu^{2+} (mg L^{-1})		
CS/LGN$_{50}$^{a}	3.12	3.21	3.09	4.12	4.18	4.04	3.63	3.70	3.56
CS/LGN$_{100}$^{b}	4.33	4.45	4.28	4.57	4.68	4.50	3.31	3.51	3.40
CS/LGN$_{150}$^{c}	4.16	4.47	4.09	7.16	7.23	7.12	5.76	5.81	5.70
CS/LGN$_{200}$^{d}	8.64	8.81	8.58	13.43	13.54	13.35	12.78	12.84	12.79
CS/LGN$_{300}$^{e}	13.59	13.71	13.51	14.44	14.56	14.36	14.32	14.14	14.22
CS/LGN$_{500}$^{f}	14.78	14.87	14.69	14.29	14.38	14.11	14.76	14.70	14.72

[a, b, c, d, e, f] sample analyzed after 50, 100, 150, 200, 300, and 500 mL of filtered solution, respectively

Adsorption experiments

The batch adsorption test for adsorption of the heavy metals ions was conducted as follows: using one set of sealed flasks, 0.02 g of aerogel was added to 50 mL of 100 ppm solution. The mixtures in the flasks were stirred to guarantee a good dispersion of the biomaterial and placed in a water bath at 25 ± 1 °C for a suitable time (12 h) to allow complete equilibration and then were filtered through filter paper. The concentrations of the initial and residual ions were determined by an inductively coupled plasma optical emission spectrometry (ICP-OES), and the results are reported in the following table S14. The removal efficiency (R) and adsorption capacity (Q, mg/g) were calculated by equation 9.

Table S 14 *Results of the batch absorption test.*

Sample	Metals		
	Pb^{2+} (mg g^{-1})	Zn^{2+} (mg g^{-1})	Cu^{2+} (mg g^{-1})
CS/LGN-CHO 1:1	28.8	27.7	31.1
CS/LGN-CHO 1.5:1	29.9	26.5	31.5
CS/LGN-CHO 2:1	30.2	26.9	32.8

Heavy metal adsorption (mg g-1) detected using aerogels as adsorbent

S11. DFT calculations

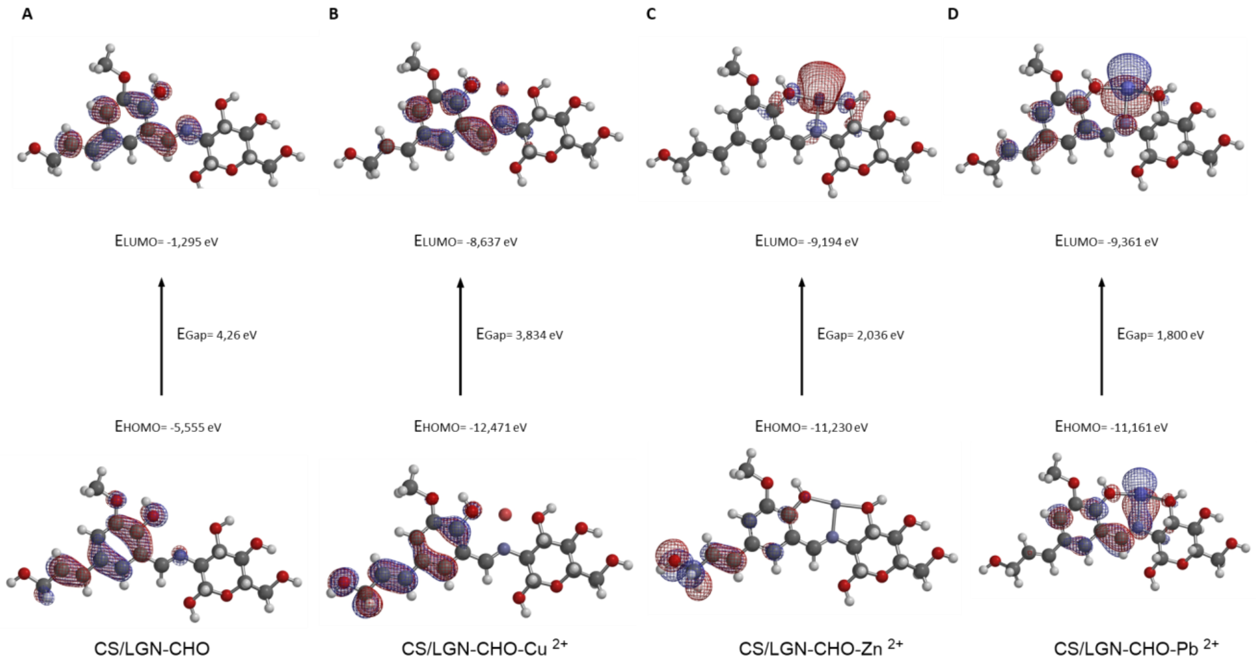

***Figure S 11**. Representation of the HOMO, LUMO and E_{gap} of: (A) CS/LGN-CHO; (B) optimized structures of the complex CS/LGN-CHO-Cu^{2+}, (C) the complex CS/LGN-CHO-Zn^{2+}, (D) the complex CS/LGN-CHO-Pb^{2+}*

***Table S 15** Second-order perturbation theory analysis of Fock matrix in NBO basis. NBO Interactions (E(2) > 1.0 kcal/mol) for Cu^{2+} complex*

Donor Index	Donor NBO	Acceptor Index	Acceptor NBO	E(2) (kcal/mol)	Energy Difference (a.u.)	F(i,j) (a.u.)
99	LP*(1) H 33	105	LP* Cu 35	20.59	0.02	0.051
89	LP (1) N 5	105	LP* Cu 35	15.90	0.54	0.118
88	LP (1) N 5	104	LP* Cu 35	15.75	0.53	0.117
90	LP (2) O 8	104	LP* Cu 35	14.46	0.73	0.132
91	LP (2) O 8	105	LP* Cu 35	13.80	0.73	0.129
96	LP (1) O 17	104	LP* Cu 35	9.91	0.73	0.111
91	LP (2) O 8	106	LP* Cu 35	9.33	0.81	0.111
90	LP (2) O 8	105	LP* Cu 35	9.28	0.81	0.112
96	LP (1) O 17	105	LP* Cu 35	7.59	0.70	0.094
88	LP (1) N 5	106	LP* Cu 35	6.28	0.54	0.077
99	LP*(1) H 33	107	LP* Cu 35	5.86	0.02	0.029
89	LP (1) N 5	107	LP* Cu 35	5.85	0.54	0.072

104	LP*(6)Cu 35	209	RY* Cu 35	5.71	0.99	0.302
105	LP*(6)Cu 35	208	RY* Cu 35	5.61	0.97	0.302
97	LP (2) O 17	105	LP* Cu 35	5.60	0.61	0.075
96	LP (1) O 17	105	LP* Cu 35	5.11	0.81	0.082
99	LP*(1) H 33	106	LP* Cu 35	4.92	0.10	0.058
96	LP (1) O 17	106	LP* Cu 35	4.41	0.78	0.075
97	LP (2) O 17	104	LP* Cu 35	3.76	0.55	0.058
96	LP (1) O 17	106	LP* Cu 35	3.11	0.74	0.061
104	LP*(6)Cu 35	208	RY* Cu 35	2.89	0.34	0.126
105	LP*(6)Cu 35	207	RY* Cu 35	2.79	0.34	0.125
97	LP (2) O 17	106	LP* Cu 35	2.49	0.69	0.053
104	LP*(6)Cu 35	216	RY* Cu 35	2.44	1.44	0.239
105	LP*(6)Cu 35	215	RY* Cu 35	2.36	1.41	0.236
96	LP (1) O 17	107	LP* Cu 35	2.22	0.70	0.050
97	LP (2) O 17	107	LP* Cu 35	1.82	0.60	0.042
62	CR (1) O 8	106	LP* Cu 35	1.51	19.21	0.218
61	CR (1) O 8	105	LP* Cu 35	1.50	19.21	0.217
18	BD (1) O 8 - H 26	106	LP* Cu 35	1.44	0.96	0.047
97	LP (2) O 17	105	LP* Cu 35	1.44	0.63	0.040
105	LP*(6)Cu 35	209	RY* Cu 35	1.40	0.64	0.123
18	BD (1) O 8 - H 26	105	LP* Cu 35	1.34	0.95	0.046
36	BD (1) O 17 - H 33	105	LP* Cu 35	1.31	1.01	0.047
104	LP*(6)Cu 35	210	RY* Cu 35	1.28	0.60	0.112
88	LP (1) N 5	105	LP* Cu 35	1.27	0.61	0.037
15	BD (1) C 7 - O 8	106	LP* Cu 35	1.27	1.01	0.046
71	CR (1) O 17	106	LP* Cu 35	1.22	19.27	0.196

15	BD (1) C 7 - O 8	105	LP* Cu 35	1.21	1.01	0.045
70	CR (1) O 17	105	LP* Cu 35	1.19	19.27	0.194
89	LP (1) N 5	208	RY* Cu 35	1.17	1.51	0.055
89	LP (1) O 8	107	LP* Cu 35	1.16	0.41	0.028
89	LP (1) N 5	106	LP* Cu 35	1.15	0.62	0.034
11	BD (1) N 5 - C 6	106	LP* Cu 35	1.15	1.04	0.044
11	BD (1) N 5 - C 6	107	LP* Cu 35	1.12	1.04	0.043
88	LP (1) N 5	209	RY* Cu 35	1.10	1.52	0.055
58	CR (1) N 5	106	LP* Cu 35	1.09	14.34	0.160
59	CR (1) N 5	107	LP* Cu 35	1.07	14.34	0.158
90	LP (1) O 8	108	LP* Cu 35	1.06	0.42	0.027

Table S 16 *Second-order perturbation theory analysis of Fock matrix in NBO basis. NBO Interactions (E(2) > 1.0 kcal/mol) for Pb^{2+} complex*

Donor Index	Donor NBO	Acceptor Index	Acceptor NBO	E(2) (kcal/mol)	Energy Difference (a.u.)	F(i,j) (a.u.)
89	LP (1) N 6	82	LP*(2)Pb 1	51.6	0.5	0.144
91	LP (2) O 9	83	LP*(3)Pb 1	21.13	0.63	0.103
96	LP (1) O 18	84	LP*(4)Pb 1	11.36	0.53	0.07
97	LP (2) O 18	83	LP*(3)Pb 1	7.27	0.39	0.048
91	LP (2) O 9	82	LP*(2)Pb 1	6.63	0.71	0.063
96	LP (1) O 18	83	LP*(3)Pb 1	6.11	0.59	0.054
96	LP (1) O 18	82	LP*(2)Pb 1	5.06	0.67	0.054
90	LP (1) O 9	84	LP*(4)Pb 1	2.26	0.29	0.023
97	LP (2) O 18	84	LP*(4)Pb 1	2.14	0.33	0.024
90	LP (1) O 9	83	LP*(3)Pb 1	1.89	0.35	0.023
14	BD (1) C 7 - H 25	82	LP*(2)Pb 1	1.51	0.6	0.028

59	CR (1) N 6	82	LP*(2)Pb 1	1.28	14.29	0.127
12	BD (2) N 6 - C 7	84	LP*(4)Pb 1	1.18	0.28	0.016
11	BD (1) N 6 - C 7	82	LP*(2)Pb 1	1.15	0.96	0.031
62	CR (1) O 9	83	LP*(3)Pb 1	1.11	19.01	0.133

Table S 17 *Second-order perturbation theory analysis of Fock matrix in NBO basis. NBO Interactions (E(2) > 1.0 kcal/mol) for Zn^{2+} complex*

Donor Index	Donor NBO	Acceptor Index	Acceptor NBO	E(2) (kcal/mol)	Energy Difference (a.u.)	F(i,j) (a.u.)
92	LP (1) N 6	84	LP*(6)Zn 1	61.68	0.66	0.183
102	LP (2) O 18	84	LP*(6)Zn 1	34.54	0.90	0.164
94	LP (2) O 9	84	LP*(6)Zn 1	32.52	0.89	0.158
94	LP (2) O 9	85	LP*(7)Zn 1	23.27	0.87	0.129
102	LP (2) O 18	85	LP*(7)Zn 1	20.96	0.88	0.123
92	LP (1) N 6	86	LP*(8)Zn 1	20.14	0.52	0.094
92	LP (1) N 6	112	RY*(2)Zn 1	12.06	2.57	0.165
92	LP (1) N 6	111	RY*(1)Zn 1	12.04	2.90	0.175
102	LP (2) O 18	112	RY*(2)Zn 1	8.90	2.81	0.145
102	LP (2) O 18	111	RY*(1)Zn 1	6.94	3.15	0.136
94	LP (2) O 9	111	RY*(1)Zn 1	6.82	3.14	0.135
94	LP (2) O 9	112	RY*(2)Zn 1	6.45	2.80	0.124
60	CR (1) O 9	85	LP*(7)Zn 1	3.31	19.26	0.230
92	LP (1) N 6	114	RY*(4)Zn 1	3.23	1.28	0.060

69	CR (1) O 18	85	LP*(7)Zn 1	3.18	19.27	0.225
92	LP (1) N 6	87	LP*(9)Zn 1	2.81	0.44	0.033
101	LP (1) O 18	87	LP*(9)Zn 1	2.81	0.41	0.031
102	LP (2) O 18	86	LP*(8)Zn 1	2.76	0.76	0.041
15	BD (1) C 8 - O 9	85	LP*(7)Zn 1	2.57	1.03	0.047
33	BD (1) C 17 - O 18	85	LP*(7)Zn 1	2.43	1.09	0.047
93	LP (1) O 9	87	LP*(9)Zn 1	2.38	0.40	0.028
95	LP (3) O 9	111	RY*(1)Zn 1	2.28	3.04	0.083
57	CR (1) N 6	86	LP*(8)Zn 1	2.27	14.31	0.163
103	LP (3) O 18	112	RY*(2)Zn 1	2.22	2.72	0.077
258	BD*(2) N 6 - C 7	87	LP*(9)Zn 1	1.97	0.05	0.025
11	BD (1) N 6 - C 7	86	LP*(8)Zn 1	1.78	1.00	0.038
14	BD (1) C 7 - H 25	84	LP*(6)Zn 1	1.70	0.77	0.034
8	BD (1) C 5 - N 6	86	LP*(8)Zn 1	1.50	0.84	0.032
102	LP (2) O 18	117	RY*(7)Zn 1	1.25	2.12	0.047
92	LP (1) N 6	119	RY*(9)Zn 1	1.18	1.95	0.045
33	BD (1) C 17 - O 18	112	RY*(2)Zn 1	1.11	3.02	0.052
94	LP (2) O 9	117	RY*(7)Zn 1	1.09	2.11	0.044
57	CR (1) N 6	84	LP*(6)Zn 1	1.05	14.45	0.118
12	BD (2) N 6 - C 7	87	LP*(9)Zn 1	1.03	0.38	0.018

92	LP (1) N 6	115	RY*(5)Zn 1	1.03	1.28	0.034

Table S18 *18 Cartesian coordinates of all investigated structures (Angstroms)*

CS/LGN-CHO-Pb^{2+}

**Atom**	**Center Number**	**X**	**Y**	**Z**
Pb	1	-0.97208	-1.94083	-0.6394
O	2	-5.13664	-0.12051	1.33527
C	3	-2.18408	2.50226	-0.15487
O	4	-1.43063	3.22015	-1.12033
C	5	-2.0744	0.98301	-0.48391
N	6	-0.73001	0.32178	-0.41221
C	7	0.38329	1.0336	-0.33994
C	8	-2.98149	0.28938	0.53662
O	9	-2.89072	-1.18373	0.34226
C	10	-4.43932	0.71581	0.38806
C	11	-4.50821	2.24019	0.66104
C	12	-5.9008	2.81592	0.38418
O	13	-6.81969	1.90979	1.08379
O	14	-3.57909	2.89476	-0.28599
C	15	4.38923	-0.20356	0.58157
C	16	3.34839	-1.13137	0.68732
C	17	2.03602	-0.76728	0.33858
O	18	1.07424	-1.81724	0.47207
C	19	1.71943	0.5385	-0.08296
C	20	2.79464	1.47079	-0.17403
C	21	4.12757	1.12385	0.13416
H	22	-6.04326	0.25728	1.48024
H	23	-1.84197	2.71529	0.8751
H	24	-1.74512	4.14657	-1.21345
H	25	0.29743	2.11412	-0.47051
H	26	-2.47119	0.86664	-1.50217
H	27	-3.69597	-1.59076	0.75683
H	28	-2.64668	0.48613	1.56289
H	29	-4.79351	0.53	-0.63853
H	30	-4.20748	2.44648	1.69953
H	31	-6.10072	2.81651	-0.69494
H	32	-5.97934	3.83812	0.77065
H	33	-7.72656	2.26905	1.15319
H	34	1.57433	-2.5841	0.87847
H	35	2.5752	2.48609	-0.49596
H	36	5.40125	-0.49929	0.8353
C	37	5.18622	2.13623	-0.02234
H	38	4.85566	3.10347	-0.39974
C	39	6.50252	1.9553	0.2361
H	40	6.89707	1.00459	0.59622
C	41	7.57083	3.0004	0.06678

H	42	7.22742	3.81992	-0.58181
H	43	7.82688	3.42282	1.05427
O	44	8.71223	2.29503	-0.50603
H	45	9.51019	2.8631	-0.54178
O	46	3.42239	-2.45262	1.11202
C	47	4.71169	-3.03728	1.54208
H	48	5.42073	-3.02737	0.70786
H	49	4.47871	-4.06324	1.82616
H	50	5.10159	-2.48512	2.40343

CS/LGN-CHO-Zn^{2+}

Atom	Center Number	X	Y	Z
Z	1	0.97381	1.89611	-0.20147
O	2	5.46209	1.18035	0.38143
C	3	2.67474	-2.00304	0.2743
O	4	1.84091	-2.98706	-0.31562
C	5	2.28759	-0.63059	-0.34526
N	6	0.91271	-0.09427	-0.14984
C	7	-0.1833	-0.82119	-0.12467
C	8	3.27731	0.37292	0.26381
O	9	2.92567	1.78028	-0.14751
C	10	4.70703	0.07643	-0.16193
C	11	5.06375	-1.32673	0.40305
C	12	6.4283	-1.81301	-0.09548
O	13	7.32959	-0.67612	0.13216
O	14	4.0571	-2.28588	-0.10283
C	15	-4.24464	0.61024	0.1463
C	16	-3.2004	1.53728	0.08966
C	17	-1.86567	1.10691	0.00811
O	18	-0.93573	2.22128	-0.0542
C	19	-1.52589	-0.26517	-0.02425
C	20	-2.60868	-1.18787	0.03049
C	21	-3.96125	-0.78407	0.1166
H	22	6.42567	0.93967	0.36413
H	23	2.59092	-1.99554	1.37619
H	24	2.20647	-3.89267	-0.20758
H	25	-0.09205	-1.90732	-0.18819
H	26	2.46003	-0.73474	-1.42579
H	27	3.7502	2.32692	-0.07417
H	28	3.2179	0.37379	1.36004
H	29	4.78922	0.04506	-1.25973
H	30	5.04757	-1.29489	1.50313
H	31	6.37383	-2.059	-1.16354
H	32	6.75172	-2.6957	0.467
H	33	8.27307	-0.91638	0.04038
H	34	-1.52821	3.03614	-0.01673
H	35	-2.38064	-2.25064	0.00034
H	36	-5.27098	0.95579	0.20139
C	37	-5.01255	-1.81431	0.1508
H	38	-4.66282	-2.84322	0.07273
C	39	-6.34326	-1.58486	0.25018

H	40	-6.75378	-0.57672	0.31629
C	41	-7.40789	-2.6465	0.28578
H	42	-7.71917	-2.81395	1.33197
H	43	-7.03587	-3.59871	-0.12058
O	44	-8.51061	-2.11072	-0.50397
H	45	-9.30859	-2.67696	-0.44711
O	46	-3.28865	2.9265	0.09583
C	47	-4.60168	3.60878	0.15923
H	48	-5.20188	3.33613	-0.71456
H	49	-4.37084	4.67368	0.14221
H	50	-5.10905	3.34536	1.0926

CS/LGN-CHO-Cu^{2+}

Atom	Center Number	X	Y	Z
O	1	5.5044	1.1204	0.5726
C	2	2.66014	-1.97677	0.19302
O	3	1.83073	-2.93303	-0.45933
C	4	2.30459	-0.56701	-0.35539
N	5	0.92404	-0.05298	-0.1586
C	6	-0.14629	-0.78097	-0.26026
C	7	3.28807	0.40673	0.31063
O	8	2.95907	1.79201	-0.09634
C	9	4.72963	0.08602	-0.07212
C	10	5.04733	-1.35267	0.41494
C	11	6.4101	-1.84725	-0.08089
O	12	7.34558	-0.76453	0.24401
O	13	4.0402	-2.2693	-0.16379
C	14	-4.26639	0.60806	0.04254
C	15	-3.24147	1.54181	-0.00558
C	16	-1.87564	1.12883	-0.12824
O	17	-0.97007	2.18725	-0.22154
C	18	-1.51822	-0.24298	-0.17298
C	19	-2.57911	-1.17162	-0.13144
C	20	-3.94886	-0.78453	-0.02503
H	21	6.45977	0.85488	0.55264
H	22	2.54697	-2.02388	1.29114
H	23	2.23675	-3.82738	-0.4403
H	24	-0.05652	-1.85938	-0.41658
H	25	2.48886	-0.60314	-1.43841
H	26	3.74755	2.36751	0.04696
H	27	3.19551	0.36892	1.40522
H	28	4.85207	0.12488	-1.16646
H	29	5.00479	-1.38385	1.51435
H	30	6.37405	-2.01839	-1.16455
H	31	6.68828	-2.77773	0.42693
H	32	8.28098	-1.02967	0.1404
H	33	-1.49124	3.03767	-0.1679
H	34	-2.34014	-2.23158	-0.17498
Cu	35	1.00491	1.99376	-0.10841
H	36	-5.29568	0.93756	0.12236
C	37	-4.9525	-1.83094	0.01449

H	38	-4.58153	-2.85369	-0.0515
C	39	-6.31214	-1.63482	0.12491
H	40	-6.74795	-0.63828	0.19438
C	41	-7.3305	-2.72716	0.15967
H	42	-7.11624	-3.41682	0.99998
H	43	-7.26252	-3.33465	-0.76476
O	44	-8.61524	-2.09347	0.29548
H	45	-9.36154	-2.72858	0.31976
O	46	-3.34621	2.91616	0.024
C	47	-4.66215	3.58815	0.13556
H	48	-5.27916	3.33526	-0.73295
H	49	-4.4351	4.65359	0.14079
H	50	-5.14613	3.29756	1.07368

Table S 19 *Atomic charges (Hirshfeld population analysis) for ONO donor atoms and metal centers in CS/LGN-CHO complexes* [a]

Complex	q(O8)	q(N5)	q(O17)	q(M^{2+})
CS/LGN-CHO–Cu^{2+}	−0.074	−0.081	−0.176	+0.79
CS/LGN-CHO–Pb^{2+}	−0.061	−0.069	−0.148	+1.21
CS/LGN-CHO–Zn^{2+}	−0.112	−0.124	−0.201	+1.58

[a] Atomic charges were obtained from Hirshfeld population analysis (with hydrogen contributions summed into the corresponding heavy atoms), as implemented in Gaussian.

References

[1] D. Gentile, G. Floresta, V. Patamia, A. Nicosia, P. G. Mineo, A. Rescifina, "Cucurbit[7]uril as a catalytic nanoreactor for one-pot synthesis of isoxazolidines in water" *Org Biomol Chem* **2020**, *18*, 1194–1203.

[2] J. J. P. Stewart, "Optimization of parameters for semiempirical methods IV: extension of MNDO, AM1, and PM3 to more main group elements" *J Mol Model* **2004**, *10*, 155–164.

[3] M. A. Matin, M. M. Islam, T. Bredow, M. A. Aziz, "The Effects of Oxidation States, Spin States and Solvents on Molecular Structure, Stability and Spectroscopic Properties of Fe-Catechol Complexes: A Theoretical Study" *Advances in Chemical Engineering and Science* **2017**, *07*, 137–153.

[4] A. A. Al-Saadi, "Computational study of SERS effects in some aliphatic and cyclic carboxylic acids with silver nanomaterials" *J Phys Conf Ser* **2020**, *1564*, 012008.

[5] Wavefunction, Inc. (2024). Spartan'24 (Version 1.2.0). Irvine, CA: Wavefunction, Inc. Available at: https://www.wavefun.com (accessed Feb 17, **2026**).

[6] X. Meng, C. Crestini, H. Ben, N. Hao, Y. Pu, A. J. Ragauskas, D. S. Argyropoulos, "Determination of hydroxyl groups in biorefinery resources via quantitative 31P NMR spectroscopy" *Nat Protoc* **2019**, *14*, 2627–2647.

[7] W. E. Benet, G. S. Lewis, L. Z. Yang, D. E. P. Hughes, “The Mechanism of the Reaction of the Tollens Reagent” *J Chem Res* **2011**, *35*, 675–677.

[8] S. Y. Lin, C. W. Dence, Eds. , *Methods in Lignin Chemistry*, Springer Berlin Heidelberg, Berlin, Heidelberg, **1992**.

[9] C. M. Hansen, *Hansen Solubility Parameters*, CRC Press, **2007**.

[10] D. Locatelli, V. Barbera, L. Brambilla, C. Castiglioni, A. Sironi, M. Galimberti, “Tuning the Solubility Parameters of Carbon Nanotubes by Means of Their Adducts with Janus Pyrrole Compounds” *Nanomaterials* **2020**, *10*, 1176.
